# Genome-Wide Association Mapping for Stripe Rust Resistance in Pakistani Spring Wheat Genotypes

**DOI:** 10.3390/plants9091056

**Published:** 2020-08-19

**Authors:** Madiha Habib, Faisal Saeed Awan, Bushra Sadia, Muhammad Anjum Zia

**Affiliations:** 1 Centre of Agricultural Biochemistry and Biotechnology, University of Agriculture, Faisalabad 38000, Pakistan; madihahabib217@gmail.com (M.H.); bushra.sadia@uaf.edu.pk (B.S.); 2 Department of Biochemistry, University of Agriculture, Faisalabad 38000, Pakistan; anjum.zia@uaf.edu.pk

**Keywords:** wheat, genome-wide association studies, association mapping, SNP, stripe rust

## Abstract

Stripe rust caused by the pathogen *Puccinia striiformis* f. sp. *tritici* (*Pst*) is a major threat for wheat, resulting in low yield and grain quality loss in many countries. Genetic resistance is a prevalent method to combat the disease. Mapping the resistant loci and their association with traits is highly exploited in this era. A panel of 465 Pakistani spring wheat genotypes were evaluated for their phenotypic response to stripe rust at the seedling and adult plant stages. A total of 765 single nucleotide polymorphism (SNP) markers were applied on 465 wheat genotypes to evaluate their stripe rust response against nine races during the seedling test and in three locations for the field test. Currently, twenty SNPs dispersed on twelve chromosomal regions (1A, 1B, 1D, 2A, 2B, 4A, 4B, 5B, 6A, 6B, 6D and 7B) have been identified that were associated with rust race-specific resistance at the seedling stage. Thirty SNPs dispersed on eighteen chromosomal regions (1A, 1B, 1D, 2A, 2B, 2D, 3A, 3B, 3D, 4B, 5A, 5B, 6A, 6B, 6D, 7A, 7B and 7D) are associated with adult plant resistance. SNP loci IWB3662 was linked with all three Pakistani races, and likewise IWA2344 and IWA4096 were found to be linked with three different USA races. The present research findings can be applied by wheat breeders to increase their resistant capability and yield potential of their cultivars, through marker-assisted selection.

## 1. Introduction

*Triticum aestivum* L., commonly known as bread wheat, is the world’s major food crop for about 2 billion people. It fulfills the nutritional requirements of almost 1/3 of the world’s population (about 35%) by providing half of their protein and above half of their calorie requirement [1]. Wheat contains a high quantity of dietary nutrients which includes carbohydrates (70%), crude fibers (22%), proteins (12%), water (12%), fats (2%) and minerals (1.8%) [2].

Wheat can withstand a wide range of climate changes but it is also prone to different biotic and abiotic stresses. Among the biotic stresses (rust, smut, mildew, bunt etc.), rust has global economic and historic significance. Rust (leaf, stripe and stem) is the most devastating fungal disease that is threatening the overall world wheat production [3] and its cyclic rotation is considered responsible for famine in many parts of the world. 

Wheat in its growing season, if faced with a cool environment, is mostly arrested by stripe (yellow) rust which is a major foliar disease caused by *Puccinia striiformis* f. sp. *tritici* (*Pst*) [4], but nowadays wheat growing in warmer regions is also prone to stripe rust epidemics [5,6]. Mostly, 0.1–5% wheat yield losses are observed due to stripe rust disease and rarely, losses expand up to 25% [4]. The yield loss due to stripe rust can exceed up to 100% for susceptible wheat landraces. Wheat infected with stripe rust causes yield loss by reducing the kernel quality, the kernel number per spike, the low-test weight and the plant height [7,8].

Pakistan is ranked among the top 10 wheat-producing countries. Globally, Pakistan ranked at seventh with 25.5 million tonnes annual wheat production [9]. In Pakistan, almost 70% of the wheat cultivation area (5.8 MH) is susceptible to stripe rust [10]. Northern and central-west areas of Pakistan are majorly threatened with stripe rust. A major yellow rust epidemic in Pakistan was observed in 1995, which caused a 20% loss in the affected areas [11]. Due to climate change, the risk factor of disease decreased but it is still at the doorstep of Pakistan. For the years 2009 and 2010, due to the high prevalence of disease in close neighbors like Uzbekistan, significant yield losses were observed. Stripe rust epidemic losses mounted to almost 360M$ in the USA for the year 2004, 100M$ in Pakistan for the year 2005 [12], 127M$ in Australia for the year 2009 [13] and 30M$ in Morocco for the year 2009 [14].

Wheat rust can be controlled by fungicides, cultural practices, and planting resistant varieties [15]. Fungicidal spray at the appropriate time can reduce the risk of loss caused by pathogens but it requires a healthy finance. Despite fungicidal spray, the areas with a high disease pressure are prevalent to substantial loss if susceptible cultivars are present [16]. In Pakistan, fungicidal control is also not a sensible choice for >8 MH area due to its high cost, spray machinery requirement, the alarming conditions of water resources, as well as air and soil pollution.

Genetic resistance is the most prevailing approach to protect the crop against the yield losses caused by diseases [17]. Resistance to stripe rust is characterized as seedling resistance (all stage resistance) and adult plant resistance (APR). All stage resistance is mostly controlled by a single resistant gene (race specific) and provides high resistance throughout the plant’s development but is readily overcome due to changes in the virulence by the emergence of new rust pathogens [18]. Alternatively, wheat resistance against stripe rust can be improved by adult plant resistance (APR), or race-nonspecific resistance or partial resistance. This nonspecific resistance is efficiently controlled by minor and effective multiple loci which are quantitatively inherited [19] and mostly appears at the later growing stages of the plant’s development [20]. Pyramiding four to five race-nonspecific resistance genes [21] makes the genotypes more durable at the later growing stages then the race-specific resistance. Gene pyramiding can be achieved by using a molecular marker closely linked with the APR and hence can be further used in breeding through marker-assisted selection (MAS). 

Molecular mapping helps in the identification of phenotypic traits at the genomic level by using molecular markers closely linked with the required traits, and hence used in marker-assisted selection and gene pyramiding. Recently, single nucleotide polymorphisms (SNPs), due to their high-density form, shown by the iSelect array, have been proven as valuable mapping markers across the whole wheat genome. The usage of SNPs in genome-wide association studies, based on linkage disequilibrium (LD), increases the efficiency of finding linked loci to the desired traits in the diverse population. Association mapping (AM) or genome-wide association studies (GWAS) utilizes the LD to spot the association among genetic polymorphism and phenotypic variation [22,23]. LD is the principle behind AM which studies the nonrandom association of alleles at different loci [22]. AM has its importance to map QTL (quatitative trait loci) due to the availability of a faster, higher density and cheaper molecular marker. It also minimizes the time and cost by utilizing the diverse populations to determine the linkage disequilibrium between the alleles and to identify the marker–trait association, over the biparental population development [24]. 

The aim of the present research was to identify the genetic divergence pattern of stripe rust resistance resources in Pakistani spring wheat genotypes. The present study also focused on the identification and mapping of seedling and field resistance minor loci for wheat stripe rust resistance using single nucleotide polymorphism (SNP) markers. 

## 2. Results

### 2.1. Phenotypic Variation to Stripe Rust Response

**Figure 1:**
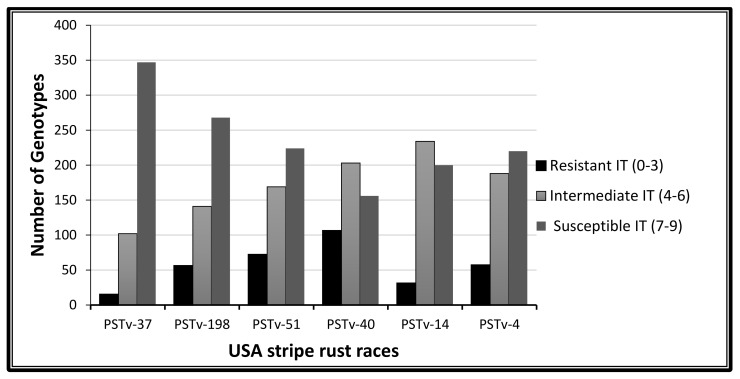
Infection distribution of the 465 spring wheat genotypes at the seedling stage based on their infection type (IT) score, tested with six stripe rust races from the USA.

**Figure 2:**
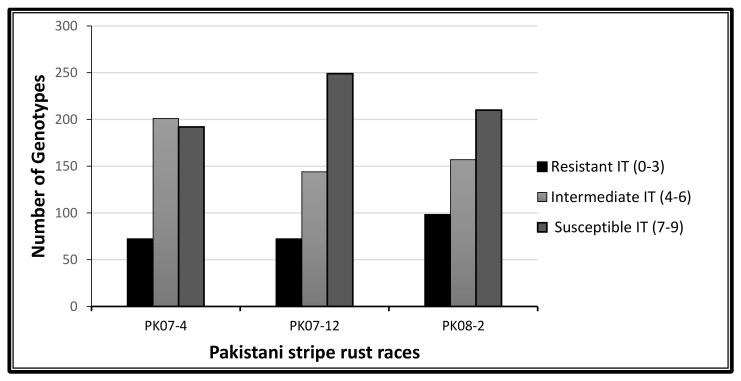
Infection distribution of the 465 spring wheat genotypes at the seedling stage based on their infection type (IT) score, tested with three stripe rust races from Pakistan.

The phenotypic characterization of 465 spring wheat genotypes was done with nine *Pst* races (six races selected from the United State of America and three races from Pakistan). The seedling test was performed in a greenhouse (controlled condition) and the rust-infection score (infection type (IT)) collected is summarized in Figure 1 and Figure 2. The frequency of resistance and susceptibility varied with all the stripe rust races. About 15, 56, 72, 106, 31 and 57 Pakistani accessions were resistant (IT score = 0–3) to the USA races PSTv-37, PSTv-198, PSTv-51, PSTv-40, PSTv-14 and PSTv-4, respectively. Most of the genotypes were susceptible (IT score = 7–9) as 384, 268, 224, 156, 200 and 220 to the USA races of stripe rust PSTv-37, PSTv-198, PSTv-51, PSTv-40, PSTv-14 and PSTv-4 respectively (Figure 1). 

Wheat genotypes showed a slightly different behavior with the Pakistani stripe rust races such that the numbers of resistant genotypes were greater when compared to the USA stripe rust races (Figure 2). The phenotypic analysis revealed 72 resistant genotypes to stripe rust races PK07-4 and PK07-12, while 98 genotypes showed a resistance response against the PK08-2 stripe rust race. Most of the genotypes were susceptible to the USA races PSTv-37 and PSTv-198 and the Pakistani race PK07-12. Most of the genotypes showed resistance to the USA races PSTv-40 and PSTv-51 and to the Pakistani race PK08-2 (Figure 1 and Figure 2).

**Figure 3:**
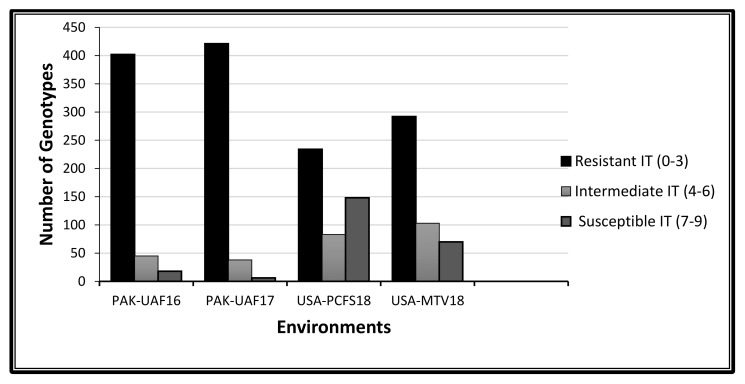
Infection distribution of 465 spring wheat genotypes at the adult plant stage and the stripe rust infection type (IT) score across three environments. Scoring for the adult plant response performed in three different environments (two years in Pakistan considered as one location and two locations in the USA).

**Figure 4:**
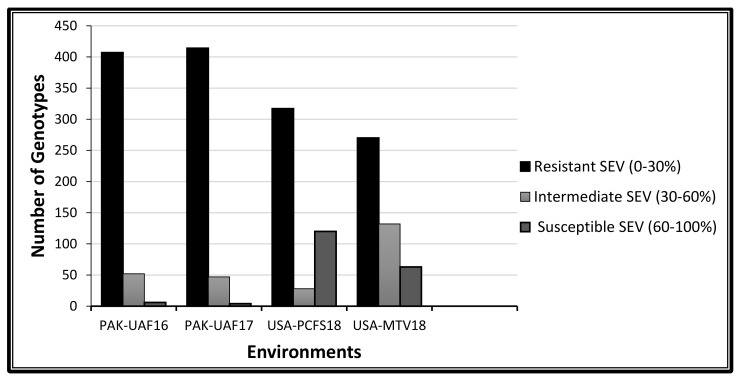
Infection distribution of 465 spring wheat genotypes at the adult plant stage and the stripe rust disease severity (SEV) score across three environments. Scoring for the adult plant response performed in three different environments (two years in Pakistan considered as one location and two locations in the USA).

In a field trial, the adult stage response of the wheat genotype against the stripe rust was slightly different as compared to the controlled conditions in the greenhouse. In a field, the resistance behavior against stripe rust infection of the genotypes was observed, namely that of PAK-University of Agriculture, Faisalabad (UAF)17 (IT = 421, SEV = 414), PAK-UAF16 (IT = 402, SEV = 407), USA-Mount Vernon (MTV)18 (IT = 292, SEV = 270) and USA-Palouse conservation field station (PCFS)18 (IT = 234, SEV = 317) with both their infection type (IT) scores and disease severity (SEV) scores (Figure 3 and Figure 4). The genotypes susceptibility frequencies were higher in the USA locations than in Pakistan, hence the response varied with the environment. In Pakistan, due to the warm environment, the disease impact was slightly less but was not negligible as compared to the cold environment near Pullman, WA, USA. The estimation of the variance component showed a highly significant behavior (*p* < 0.0001) of the genotypes and the genotype x environment interaction across all the environments. Non-significant responses of environmental variance indicate the variable climate conditions and the influence of different races in the disease development. A high range of the coefficient of determination and broad sense heritability of (H^2^) 87% for the IT and 93% for the SEV indicate the reliability of the dataset for GWAS (Table 1).

### 2.2. Population Structure and Linkage Disequilibrium

**Figure 5:**
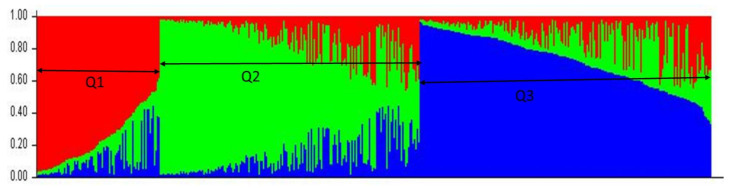
Estimated population structure of the 465 spring wheat genotypes (K = 3) based on the Q matrix using the single nucleotide polymorphism (SNP) markers. Q: three (K = 3) different subpopulations as Q1, Q2 and Q3.

The population structure was performed on a total 465 entire wheat genotypes by the Bayesian clustering approach. An admixture-based model was used to cluster the genotype into three subpopulations based on the ΔK (Figure 5; Supplementary Table S4) value. Population structure reduces the false positive association among the markers and traits. Subpopulation one (Q1) contains 85 individuals. Similarly, subpopulation two (Q2) contains 179 and subpopulation three (Q3) contains 201 individuals. Each subpopulation is shown by a different color in the cluster analysis. The length of each color represents the estimated contribution of each sample to the subgroups.

**Figure 6:**
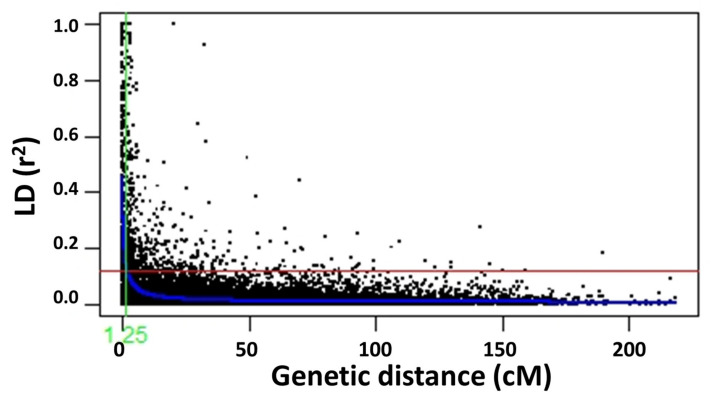
Scatter plot of the linkage disequilibrium (LD) decay with the critical *r*^2^ value and the genetic chromosome distance (cm) for the whole genome. The red line shows the critical *r*^2^ value i.e., 0.12.

After the filtration of minor allelic frequency, 765 SNP markers out of 1500 SNPs were used for the linkage disequilibrium analysis (LD). Linkage disequilibrium (LD) depends on many factors, including the population structure, genetic drift, chromosomal region and natural selection. LD decay relies on the value of *r*^2^, whose value is calculated for all chromosomes. Critical *r*^2^ value 0.12 was identified for all 465 spring wheat genotypes by taking the 95th percentile of the coefficient square (showed by the red line in Figure 6). The mean *r*^2^ value across the genome was found 0.03 with 50 cm distance. The highest number of pair-markers were found on the A genome (48%) followed by the B genome (46%) and the D genome (5%). Chromosome 1A had the highest number of pairs (2138) and 4D had the minimum number (nine) of pair markers. The LD decay was constructed using chromosomal distance and the critical *r*^2^ value as the threshold to indicate the LD decay length, which attained 1.25 cm for the whole genome (Figure 6). 

### 2.3. Marker–Trait Association at Seedling Stage

Genome-wide association studies (GWAS) of SNP markers with IT scores of nine stripe rust races identify twenty SNPs associated with rust resistance at the seedling stage (Table 2). Manhattan and Q-Q plots, showing the marker–trait association of stripe rust-resistance response at the seedling stage, with all nine races IT scores are provided in the Supplementary Figures S1 and S2. Twelve chromosomal regions (1A, 1B, 1D, 2A, 2B, 4A, 4B, 5B, 6A, 6B, 6D and 7B) were found to be significantly associated (*p* < 0.0001) with the IT scores of nine rust races at the seedling stage. A total of fourteen SNP loci were identified to be linked with resistance against USA races and twelve loci were identified to be linked with stripe rust-resistance response to Pakistani races. One SNP present on chromosome 1A (IWB3662) was found to be associated with all three Pakistani rust races, namely PK07-4, PK07-12 and PK08-2. A SNP on the 1B chromosome (IWB12258) was linked with the IT score of PK08-2. At the chromosome 1D, two SNPs IWA7171 (linked to PK07-12) and IWA4344 (linked to PSTv-4) were identified. Two SNP markers, IWB50806 linked with PK07-12 and IWA7638 linked with PSTv-14, were identified on chromosome 2A. The targeted SNP IWA2344 was associated with three rust races PSTv-198, PSTv-51 and PSTv-40 at chromosome 2B. Similarly, for the three rust races, PSTv-198, PSTv-51 and PSTv-4, one SNP (IWA4097) marker on the chromosome 1B was identified. Two SNPs were identified at chromosome 2B associated with six stripe rust race. At chromosome 4A, one SNP (IWA3361) identified associated with PSTv-4. Three SNPs identified on the 4B chromosome, IWB12434, IWA2031 and IWA408, were associated with PK07-12, PSTv-14 and PK08-2, respectively. The SNP IWB5781 was associated with PSTv-4, identified at chromosome 5B. At 6A three SNPs identified IWA8022 linked with PSTv-40 and IWA3463, IWB25252 both linked with PK07-12. One SNP (IWB7615) on chromosome 6B was associated with PSTv-51. IWB12259 was linked with PK07-4 at the location 6D. Finally, two SNPs (IWA6857 and IWB10895) were associated with PK07-12 and PSTv-40 on chromosome 7B. Seven SNPs were identified at genome A, ten SNPs at genome B and three SNPs at genome D. The largest number of SNPs (six) was identified to be linked to PK07-12. The identified SNP markers associated with rust race-specific resistance can be linked to different Yr genes based on the virulence and avirulence formula of races (Supplementary Table S2 and S3).

### 2.4. Marker–Trait Association at Adult Plant Stage

Genome-wide association studies of 765 SNPs with the IT and SEV responses of 465 wheat genotypes against stripe rust at three different locations were performed. The association with the IT scores at different locations, namely PAK-UAF16, PAK-UAF17, USA-MTV18, USA-PCFS18 and with the BLUE (best linear unbiased estimator) value identifies twenty-two SNPs that were associated with stripe rust resistance at the adult plant stage. Significant SNPs was identified based on *p* < 0.001. A total of fifteen chromosomal regions (1A, 1B, 1D, 2A, 2B, 2D, 3A, 3B, 4B, 5A, 5B, 6A, 6D, 7A and 7B) were mapped with the identified SNPs. The highest number of SNPs with an IT score was identified in the USA environment, whereas with the SEV response, twenty-two SNPs were mapped in sixteen chromosomal regions (1A, 1B, 1D, 2A, 2B, 2D, 3A, 3B, 3D, 4B, 5A, 6A, 6B, 7A, 7B and 7D). Overall, thirty significant SNPs were identified with both IT and SEV response. The highest number of loci (eight) was mapped for the 2A chromosomes with the SNPs IWA11136, IWB12554 and IWA6798 at 9.41 cm, 143.6 cm and 150.1 cm respectively. The SNP name, chromosome, position and the probability of its association with IT and SEV, are discussed in detail in Table 3. Five chromosome regions (1D, 3D, 4B, 6D and 7A) were identified that were mapped with significant SNPs in the Pakistan environments with both the IT and SEV disease scores of the genotypes. Nine regions were identified with both the IT and SEV response in the environment USA-MTV18 and eight regions were mapped with significant SNP markers in the environment USA-PCFS18 (Table 3). In the present work, the SNP marker, IWB11136 (2A at 9.41 cm), was found to be linked with all the USA locations with both IT and SEV resistant scores of stripe rust. Manhattan and Q-Q plots showing the marker–trait association with the stripe rust resistance response IT and SEV scores, at all three locations and with the BLUE value, are provided in Supplementary Figures S3 and S4.

## 3. Discussion

### 3.1. Phenotypic Variation to Stripe Rust Response

The present study was conducted to study the spectrum of genetic diversity, as well as to map the resistance loci in 465 Pakistani spring wheat genotypes in response to stripe rust (*Puccinia striiformis* f. sp. *tritici*) at seedling and adult plant stage. Profiling with high-density SNP markers helps in the identification of genomic diversity, population structure among genotypes and marker–trait association to identify well-worn and novel resistance sources in germplasm. In previous reports, variable numbers of stripe rust races were used to compute the response against the specific race [19,26,27,28]. In the current study, a total of nine *Pst* races, including six from the USA and three of Pakistani origin were used to evaluate the genotypes response at the seedling stage also three different locations, tested for adult plant response (two years in Pakistan considered as one location and two locations in the USA). 

In the current study, almost 50% intermediate response was observed at the seedling stage. Moreover, a high level of resistance (IT score = 0–3) response appeared at the adult plant stage and a low level of resistance phenotype appeared at the seedling stage. Similarly, a more susceptible response appeared at the seedling stage than at the adult plant stage [16,27,28,29]. Wheat genotypes were more susceptible to USA stripe rust races compared to Pakistani stripe rust races, where the resistant phenotype response was dominant. Pakistani wheat genotypes have long history of cultivation [30] before the emergence of modern wheat. This suggests that they have more interaction with *Pst* races, prevailing themselves as a major source of resistance to *Pst* [31].

Population structure, based on the Bayesian model, divides the 465 genotypes into three subpopulations (K = 3). Based on the genotype diversity, a different number of subpopulations were reported, as two subpopulations [16], three subpopulations [29], six subpopulations [32], seven subpopulation [31] and eight subpopulations [27]. Critical *r*^2^ was used to estimate the extent of the LD decay with the line intersecting the smooth curve [19]. In present study, the critical *r*^2^ value for the whole genome was 0.12 (for 765 SNPs) and we found LD decay at a confidence interval of 1.25, previously reported as 1.6 cm [31].

### 3.2. Genome-Wide Association Analysis with Stripe Rust Response

The major factors that influenced the association mapping were the population size, the germplasm choice and the marker density over whole genome [32]. Several association mapping studies were reported for stripe rust using different molecular markers as diversity arrays technology (DArT) [26,33,34], simple sequence repeats (SSR) [35,36] and SNP [19,27,29,37,38]. 

Marker–trait association at the seedling stage with nine different races identified twenty QTLs covering twelve chromosomes (1A, 1B, 1D, 2A, 2B, 4A, 4B, 5B, 6A, 6B, 6D and 7B) with *p* < 0.0001. The SNP loci *QYr.uaf-1D.2* associated with stripe rust resistance at the seedling stage was also found to be associated with rust resistance at the adult plant stage. 

SNP loci *QYr.uaf-1A.1*, mapped on the short arm of 1A positioned at 10.69 cm, was found to be associated with resistance to all three Pakistani races (PK07-4, PK07-12 and PK08-2). Likewise, earlier it was reported that the position of SNP IWB3662 lay within the confidence interval of *YrEDWL-1AS,* associated with resistance to PSTv-14 and PSTv-37 in durum wheat [19] and *QYrid.ui-1A_Rio* Blanco [39]. Three SNP markers (IWB12795, IWB20633 and IWB56353) associated with the loci *QYr.tsw-1A* were positioned on the 1A chromosome had already been reported [16] in close proximity to currently identified loci that were linked with resistance to PSTv-4 in spring wheat germplasm. Stripe rust-resistant locus *QYr.uaf-1B.1* tagged with SNP IWB12258 in current research findings was found in close proximity of IWA1191 [28]. The Chromosomal position 1B reported with many *Yr*-associated genes as *Yr9, Yr10, Yr15, Yr34/Yr26, Yr29, Y64, Yr65, YrAlp* and *YrH52* [31]. Chromosome 1B locus *QYr.uaf-1B.1* was found in close proximity to *QYrcau-1BS_AQ24788-53* [40] from Chinese winter wheat and *Yr9* resistant gene [31]. In Pakistan, *Yr9* was first reported in 1994, and after that many cultivated varieties were developed with this gene, due to its linkage with other genes (*Lr26, Sr31, Pm8*) and pleiotropic effect [41]. The successful translocation of the *Yr9* gene from rye and alongside high-yield potential, made *Yr9* a highly dominated gene in Pakistani germplasm [35]. Wheat varieties carrying this gene were highly cultivated during 1990s, but resistance breakdown after a few years made the resistant cultivars susceptible [42]. A major yellow rust epidemic was observed in 1995 with a 20% loss in the affected area in Pakistan. At that time, Pak81, also known as Veery#5 carrying *Yr9* gene, predominated. Two major cultivars, Pak81/Pirasabak 85 became susceptible during the period 1994–1995 due to the ineffectiveness of the *Yr9* gene and Inquilab 91 in 2002 due to the virulence occurrence of the *Yr27* gene [11]. 

Single stripe rust resistant QTL, *QYr.tsw-1D,* against the USA race Pstv-14, as reported by [16], was found in close proximity to SNP IWA7171 and IWA4344, positioned at 90.30 cm on the 1D chromosome. QTL *QYr.uaf-1D.1,* was linked with resistance to PK07-12 and PSTv-4 in Pakistan’s spring wheat population. SNP loci *QYr.uaf-2B.1* (IWA2344) was positioned on the 2B at 96.99 cm, was found to be linked to all-stage resistance with the three stripe rust races PSTv-198, PSTv-51, PSTv-40, and was found in the confidence interval of *QYr.ucw-2B_UC1110* [43], *QYr.inra-2B.1_Camp* Remy [44] and *QYr.cim-2BS*_*Francolin* [45]. It was found that the second identified loci of chromosome 2B (*QYr.uaf-2B.2*) positioned at 113.86 cm was also associated with the three rust races PSTv-198, PSTv-51 and PSTv-4. The locus (*QYr.uaf-2B.2*) was found in the confidence interval of *QYraq.cau-2BL_Aquileja* [46] and *Yr53* [47]. 

Three QTLs *QYr.uaf-4B.1, QYr.uaf-4B.2, QYr.uaf-4B.3* at chromosome 4B were found in close proximity to *QYr.jic-4B_Alcedo* [48], *QYr.jic-4B_Guardian* [49], *QYr.vt-4BL_VA00W-38* [50] and *YrEDWL-4BL,* in novel durum wheat linked to the stripe rust race PSTv-14 and PSTv-51 [19]. One QTL *QYr.uaf-5B.1* on the 5B chromosome positioned at 56.6 cm, was found within the confidence interval of *QYr.ufs-5B_Cappelle-Desprez* [51]. Two SNP markers (IWA3463, IWB25252) linked the QTL *QYr.uaf-6A* to stripe rust resistance, and PK07-12 was found in close proximity to the previously reported QTL *QYr.cim-6AL_Francolin* [45]. *QYr.uaf-7B.1* and *QYr.uaf-7B.2* were found to be linked with resistance to the stripe rust races PK07-12 and PSTv-40, respectively, and were found to be linked to the *QYr.sun-7B_Kukri* [52], *Yr39* gene [53] and near to the marker IWA312 (76.1 cm) that was linked with resistance to the *Pst* races Pstv-37 and Pstv-40 [28].

### 3.3. Genome-Wide Association Analysis for Adult Plant Response

Genome-wide association (GWA) analysis of stripe rust using high-density SNP markers was carried out across the three locations. In total, thirty loci were identified that were significantly (*p* < 0.001) associated with the infection type (IT) and disease severity (SEV) score of stripe rust in multi-environments. These thirty loci were present in eighteen genomic regions, namely 1A, 1B, 1D, 2A, 2B, 2D, 3A, 3B, 3D, 4B, 5A, 5B, 6A, 6B, 6D, 7A, 7B and 7D. In the current study, three loci were mapped on the chromosomes 1A, 2A, 3A and 3B. Two QTLs were identified on each chromosome 1D, 4B, 5A and 7B. One QTL was identified on each chromosome 1B, 2B, 2D, 3D, 5B, 6A, 6B, 6D, 7A and 7D. 

In the present research work, the QTL, *QYr.uaf-2A.3* (linked SNP IWB11136), identified to be positioned at 9.41 cm on the 2A chromosome, was significantly associated with six locations including the IT (USA-MTV18, USA-PCFS18, BLUE-IT) and SEV response (USA-MTV18, USA-PCFS18 and BLUE-SEV). The same SNP marker IWB11136 (*QYr.tsw-2A.3*) that was identified previously was also found significantly associated with all the tested locations for the stripe rust response [16]. This locus was also found within the confidence interval of *QYr.tam-2AS* [54] from the hard winter wheat TAM111 and *Yr17* genes [55]. The *Yr17* gene was developed by 2NS–2AS locus translocation (25 to 38 cm) from *T. ventricosum* (2NS), a famous wild *Triticeae* species to 2AS of wheat chromosome [55,56]. The 2NS–AS translocation was first carried out in winter bread wheat VPM1 and afterwards in California and Washington, where many winter wheat cultivars were developed such as Madsen, Hyak and Expresso (spring wheat). Furthermore, the 2B QTL *QYr.uaf-2B.3* positioned at 130.6 cm was linked with both the IT and SEV score at the USA location MTV, and was linked with the *Yr* genes *Yr53* and *Yr43* [47]. At chromosome 3B, two SNP markers (IWB11270 and IWB36652) were associated with the QTLs *QYr.uaf-3B.2* and *QTL QYr.uaf-3B.3*, and were aligned with both the IT and SEV scores of USA-MTV, USA-PCFS18 and the BLUE value, which was found in close proximity to *QYr.cim-3B_Pastor* and *QYr.inra-3Bcentr_Renan* [57,58]. 

Presently, two QTLs *QYr.uaf-3B.2* (IWB11270) were positioned at 67.67 cm on 3B and *QYr.uaf-5A.2* (IWA589) was positioned at 123.21 cm on chromosome 5A, which was significantly associated (*p* < 0.001) with stripe rust resistance at four different locations. Locus *QYr.uaf-3B.2* was found in the confidence interval of *QYr.cim-3B_Pastor* [58], whereas the QTL locus *QYrEDWL-5AL.2* reported in Ethiopian durum wheat was in close proximity to *QYr.uaf-5A.2* [19]. *Qyr.wpg-6B.1* (IWA7257) in winter wheat and *QYr.cim-6BL_Pastor* was found in close proximity *to QYr.uaf-6B.2* (IWB26626) positioned at 113.67 cm on the chromosome 6B [37,57]. Five SNPs loci identified in the current study were linked with resistance to either the IT or SEV response of the stripe rust at three different locations. These included *QYr.uaf-1B.2*, *QYr.uaf-1D.2*, *QYr.uaf-2B.3*, *QYr.uaf-4B.2* and *QYr.uaf-5A.1*. The identified resistance source can be utilized as the breeding line for enhancing wheat resistance against disease.

## 4. Materials and Methods

### 4.1. Collection of Genetic Material

Four hundred and sixty-five (465) genotypes of bread wheat (*Triticum aestivum* L.) were collected from the Wheat Research Institute, Ayub Agricultural Research Institute (AARI), Pakistan, Faisalabad. 

### 4.2. Field Based Resistance Screening to Puccinia striiformis (Pst)

The panel of 465 spring wheat genotypes was tested for stripe rust response under field conditions. All genotypes were grown at the experimental area of the Centre of Agricultural Biochemistry and Biotechnology (CABB), University of Agriculture, Faisalabad (31°26′ N, 73°6′ E) for two consecutive years 2015–2016 and 2016–2017. Each entry was planted in a 1 m long row by keeping a row-to-row distance of 36 cm and the sowing was done by planting two seeds per hole and maintaining 8 cm plant-to-plant distance. The highly susceptible variety of stripe rust i.e., Morocco was used as the susceptible check in the field experiments in Pakistan to increase the disease pressure. All 465 Pakistani wheat genotypes were also sown at two different locations in the USA at the Palouse conservation field station (PCFS), Pullman, WA (46°43′59″ N; 117°10′19″ W) and Mount Vernon (MTV), WA (48°25′16″ N; 122A°20′2″ W) in the year 2018 for the evaluation of stripe rust resistance under natural disease conditions. Spring wheat Avocet susceptible (AvS) was used as the susceptible check to increase the disease pressure at both locations planted after every twenty lines in the USA. Each line of the genotype was grown up in a 0.5 m long and a 0.3 m wide row. All standard agronomic practices were followed for the crop production. 

The evaluation of *Pst* at the adult plant stage was done in a field with the natural conditions of disease epidemics at all three locations (two years in Pakistan considered as one location and two locations in USA). In the field, the data scoring was done by visualizing the impact of the disease on the flag leaf of the susceptible check that the IT score, which ranged from 7–9 and the disease severity score which ranged from 70–100% [19]. One location was in Pakistan at the University of Agriculture, Faisalabad (UAF) for two consecutive years 2015–2016 (PAK-UAF16) and 2016–2017 (PAK-UAF17). The other two locations data was recorded at the USA for year 2018 at Pullman, WA (USA-PCFS18) and at Mount Vernon, WA (USA-MTV18). Infection type (IT) and disease severity (SEV) were two disease phenotype scores that were recorded for the *Puccinia striiformis* (*Pst*) infection in the field. The IT score based on the 0–9 scale is explained in Supplementary Table S1 whereas the SEV recorded as the % age area of the flag leaf covered with disease and was scored from 0 to 100%. 

### 4.3. Greenhouse-Based Resistance Screening to Puccinia striiformis (Pst)

The seedling response of 465 wheat genotypes was evaluated with isolates of six *Pst* USA races including PSTv-37, PSTv-198, PSTv-51, PSTv-40, PSTv-14, PSTv-4 [25] and three isolates of Pakistani races PK07-4, PK07-12, PK08-2 [59] under controlled greenhouse conditions. The virulence and avirulence formulae of the stripe rust race isolates are provided in the Supplementary Tables S2 and S3. All the stripe rust races were collected from the USDA, Wheat Health, Genetics and Quality Research Unit, Pullman, WA. Four to five seeds of each genotype were planted per well in a 96 wells tray filled with the number 1 sunshine mix growing medium (Sungro Horticulture, Bellevue, WA, USA). Trays were regularly watered and kept in a rust free greenhouse at 20 °C with 50% relative humidity (RH). The inoculation was done when the seedling reached the 2-leaf stage after approximately 9–10 days of sowing. The inoculation of each race was done by mixing the rust urediniospores with talcum powder. Inoculated plants were incubated in a dark dew chamber for 24 h at 10 °C and 100% relative humidity and then moved to the greenhouse having the 8 °C day, 16 °C night temperature and 16 h photoperiod. The reaction to *Puccinia striiformis* f. sp. *tritici* was scored after 18 to 20 days of inoculation, using the 0–9 scale for the infection type (IT) [60,61]. Based on the infection type, the genotypes were grouped as 0–3 = resistant; 4–6 = intermediate; 7–9 = susceptible. The scale of the infection type (IT) disease score is discussed in Supplementary Table S1.

### 4.4. Statistical Analysis

The range, mean, standard deviation, coefficient of determination (R^2^) were scored within and across the environments using JMP Genomics 15.1.0 (SAS Institute Inc., Cary, NC, USA, 2007). Broad sense heritability (H^2^) was calculated by the variance component obtained from REML (random effects model) computed using JMP software. The BLUE (best linear unbiased estimator) value for the IT and SEV scores of all environments was calculated using the PROC MIXED procedure in SAS v9.3 (SAS Institute Inc., Cary, NC, USA, 2007) considering the genotype as a fixed effect [16].

### 4.5. DNA Extraction, SNP Genotyping

Wheat genotypes sown in greenhouse and young leaves used for DNA extraction using a robotic system of oKtopureTM at the Western regional small grain genotyping laboratory (WRSGGL) (Washington State University, Pullman, WA, USA) [62] for SNP genotyping against stripe rust. Targeted amplicon sequencing (TAS) for stripe rust resistant genes was done using NextSeq^®^ 500 (Illumina, Inc., Pullman, WA, USA). Genotypic calling and removing monomorphic as well as low quality SNPs, was carried out using GenomeStudio Software v2011.1. (Illumina, Inc., Pullman, WA, USA) to call bi-allelic SNPs AA, AB and BB, for this default clustering algorithm, was used. A total 1500 SNPs were yielded and subjected to TASSEL (trait analysis by association, evolution and linkage) software v.5.2.61 [63] to remove the SNPs with minor allelic frequency MAF < 0.05 and to made kinship matrix. A total 765 high-quality SNPs were selected and projected on to a consensus map of hexaploid wheat to order them based on the chromosome position [25]: these 765 SNPs were used for association analysis. 

### 4.6. Population Structure and Linkage Disequilibrium (LD)

Major genetic structure of the selected genotypes was determined using 150 SNP markers with inter marker distances >5 cm from each other using the Bayesian model-based clustering algorithm in STRUCTURE v2.3.4 [64]. The number of subpopulations (K) was estimated by the running simulation from burn-in 10,000 iteration to 100,000 Monte Carlo Markov Chain (MCMC) replicates. K was run from 1–10 times and 10 independent runs were set for each run. The STRUCTURE results were visualized to determine the value of K (subpopulation) based on the ad hoc criterion by using the STRUCTURE HARVESTER [65,66]. 

The measurement of the linkage disequilibrium between the pairs of the SNP marker was estimated using the program TASSEL (v5.2.61). The LD parameters D’ and *r*^2^ among the loci and comparison-wise significance was computed by 1000 permutations. The critical *r*^2^ value was determined by taking the 95th percentile of the unlinked markers [67]. The scatter plot among the *r*^2^ and distance on chromosome, of all significant (*p* < 0.001) pairwise combinations, were used to fit the locally weighted polynomial regression curve (LOESS) to estimate the extent of the LD decay in the R environment [16] using the critical *r*^2^ value. 

### 4.7. Genome-Wide Association (GWA) Analysis

Integrated mixed-model (MLM) method for association mapping, which accounts for multiple levels of relatedness, was used to narrate the genetic polymorphism to important phenotypic variation in specific traits [68]. An association test was performed using both (1) the Genome Association and Prediction Integrated Tool (GAPIT) [69] and (2) fixed and random model circulating probability unification (FarmCPU) package [70] implemented in R software v.3.6.1 (https://www.r-project.org/). The MLM model utilized trait data, genotype data K (kinship) and PCA (principle component analysis) to find the marker–trait association. The model comparison was done to select the best model for the marker–trait association (MTA) with each trait as K + P (kinship and principal component) [44] and K + Q (kinship and population structure) [68]. The final results were analyzed by FarmCPU, selecting the model (K + Q) based on their respective Q-Q plots. Significant MTA was described based on the *p*-value. Markers with *p* < 0.0001 were considered significant for the seedling test and *p* < 0.001 for the field experiment. Marker–trait association was performed with all nine rust races data, scored at the seedling stage and with the field data of all the environments separately for the IT score, the disease severity score and with the best linear unbiased estimator (BLUE) value using the FarmCPU package implemented in R software v.3.6.1. 

## 5. Conclusions

GWAS provides a good outline of the distribution and frequency of resistance genes over the whole world subpopulation. This spring wheat Pakistani germplasm was proved an efficient source of phenotypic diversification to combat stripe rust infection for both seedling and field experiments and to determine the yield QTLs related to the yield components. The genotypes possessing a higher fraction of resistance loci of stripe rust divulged themselves as a parental breeding line and hence can increase the breeding efficiency for stripe rust resistance. The present research findings can be exploited by wheat breeders to increase the resistant capability and yield potential of their cultivars by marker-assisted selection breeding.

## Figures and Tables

**Table 1:** Mean, range and the variance components of *Puccinia striiformis* under different environments.

Parameter	PAK-IT	PAK-SEV	USA-IT	USA-SEV	ALL-IT	ALL-SEV
**Range**	0-8	0-80	2-8	1-100	0-8	0-100
**Mean**	2.14	12.32	4.17	30.85	3.15	21.53
**Var(G)**	1.529 **	175.66 **	3.035 **	440.97 **	0.962 **	119.39 **
**Var(E)**	0.006 ^ns^	0.238 ^ns^	0.3 ^ns^	0.303 ^ns^	1.47 ^ns^	114.26 ^ns^
Var(G*E)	0.446 **	25.54 **	2.094 **	447.7 **	2.64 **	433.91 **
**Var(Total)**	1.98	201.44	5.429	888.98	5.07	667.57
**R^2^**	0.87	0.93	0.76	0.687	0.54	0.43
**SD**	1.418	14.37	2.38	30.93	2.21	25.8
**H^2^**	87	93	71	66	48	46

Asterisks ** significant at *p* < 0.0001 and * significant at *p* < 0.005, **ns**: non-specific, **IT**: infection type, **SEV**: disease severity, **Var**: variance, **R^2^**: coefficient of determination, **SD**: standard deviation, **H^2^**: broad sense heritability.

**Table 2:** SNPs associated with the seedling response to nine *Pst* races in 465 genotypes of wheat (*p* < 0.0001).

QTL ^a^	SNP Id. ^b^	Chr. ^c^	Position ^d^ (cm)	MAF ^e^	−log_10_(P) ^f^								
					PK07-4	PK07-12	PK08-2	PSTv-37	PSTv-198	PSTv-51	PSTv-40	PSTv-14	PSTv-4
*QYr.uaf-1A.1*	IWB3662	1A	10.69	0.46	8.14	9.03	6.59	_	_	_	_	_	_
*QYr.uaf-1B.1*	IWB12258	1B	28.76	0.24	_	_	4.77	_	_	_	_	_	_
*QYr.uaf-1D.1*	IWA7171	1D	90.30	0.30	_	4.57	_	_	_	_	_	_	_
*QYr.uaf-1D.2*	IWA4344	1D	90.30	0.21	_	_	_	_	_	_	_	_	4.69
*QYr.uaf-2A.1*	IWB50806	2A	81.49	0.47	4.42	_	_	_	_	_	_	_	_
*QYr.uaf-2A.2*	IWA7638	2A	162.89	0.46	_	_	_	_	_	_	_	4.3	_
*QYr.uaf-2B.1*	IWA2344	2B	96.99	0.26	_	_	_	_	6.91	6.09	5.55	_	_
*QYr.uaf-2B.2*	IWA4097	2B	113.86	0.10	_	_	_	_	8.36	7.53	_	_	5.32
*QYr.uaf-4A.1*	IWA3361	4A	48.84	0.24	_	_	_	_	_	_	_	_	4.66
*QYr.uaf-4B.1*	IWB12434	4B	74.62	0.46	_	4.32	_	_	_	_	_	_	_
*QYr.uaf-4B.2*	IWA2031	4B	98.65	0.16	_	_	_	_	_	_	_	4.51	_
*QYr.uaf-4B.3*	IWA408	4B	114.87	0.30	_	_	4.23	_	_	_	_	_	_
*QYr.uaf-5B.1*	IWB5781	5B	56.60	0.47	_	_	_	_	_	_	_	_	4.19
*QYr.uaf-6A.1*	IWA8028	6A	77.14	0.27	_	_	_	_	_	_	4.06	_	_
*QYr.uaf-6A.2*	IWA3463	6A	83.04	0.34	_	4.02	_	_	_	_	_	_	_
*QYr.uaf-6A.3*	IWB25252	6A	95.87	0.26	_	4.28	_	_	_	_	_	_	_
*QYr.uaf-6B.1*	IWB7615	6B	64.82	0.43	_	_	_	_	_	4.81	_	_	_
*QYr.uaf-6D.1*	IWB12259	6D	155.10	0.18	4.51	_	_	_	_	_	_	_	_
*QYr.uaf-7B.1*	IWA6857	7B	76.31	0.08	_	5.4	_	_	_	_	_	_	_
*QYr.uaf-7B.2*	IWB10895	7B	103.21	0.45	_	_	_	_	_	_	4.07	_	_

^a^ Putative name of the identified quantitative trait loci (QTL) from the marker–trait association, ^b^ SNP marker associated with all stage resistance to stripe rust against the different races, ^c,d^ Chromosome number and position of the associated SNP marker according to [25], ^e^ MAF: Minor allelic frequency, ^f^ *p*-value of marker–trait association.

**Table 3:** SNPs associated with adult plant response (IT and SEV) at three locations with *p* < 0.001.

QTL ^a^	SNP Id. ^b^	Chr. ^c^	Pos. ^d^ (cm)	MAF ^e^	−log10(p) Based on IT ^f^ Score					−log10(p) Based on SEV ^g^ Score				
					PAK-UAF16	PAK-UAF17	USA-MTV18	USA-PCFS18	BLUE-IT	PAK-UAF16	PAK-UAF17	USA-MTV18	USA-PCFS18	BLUE-SEV
*QYr.uaf-1A.2*	IWB3662	1A	10.69	0.45	_	_	_	_	_	_	_	_	_	3.24
*QYr.uaf-1A.3*	IWA2541	1A	95.55	0.22	_	_	_	_	3.52	_	_	_	_	_
*QYr.uaf-1A.4*	IWA4080	1A	96.3	0.5	_	_	4.03	_	_	_	_	_	_	_
*QYr.uaf-1B.2*	IWA5278	1B	70.08	0.34	_	_	_	3.46	_	_	_	_	3.34	3.47
*QYr.uaf-1D.3*	IWA642	1D	67.72	0.32	_	_	_	3.16	_	_	_	_	_	_
*QYr.uaf-1D.2*	IWA4344	1D	90.3	0.21	3.22	_	_	_	3.27	3.08	_	_	_	_
*QYr.uaf-2A.3*	IWB11136	2A	9.41	0.36	_	_	4.71	3.25	3	_	_	4.25	3.03	3.23
*QYr.uaf-2A.4*	IWB12554	2A	143.65	0.28	_	_	_	_	_	_	_	3.04	_	_
*QYr.uaf-2A.5*	IWA6798	2A	150.11	0.4	_	_	_	_	_	_	_	3.29	_	_
*QYr.uaf-2B.3*	IWA3478	2B	130.62	0.31	_	_	5.19	_	_	_	_	5.7	_	3.48
*QYr.uaf-2D.1*	IWA5673	2D	82.82	0.16	_	_	3.32	_	_	_	_	3.32	_	_
*QYr.uaf-3A.1*	IWA288	3A	49.1	0.32	_	_	_	3.59	_	_	_	_	_	_
*QYr.uaf-3A.2*	IWB73771	3A	66.48	0.37	_	_	_	4.24	_	_	_	_	_	_
*QYr.uaf-3A.3*	IWB68593	3A	195.1	0.36	_	_	_	_	_	_	_	3.6	_	_
*QYr.uaf-3B.1*	IWB11085	3B	25.09	0.15	_	_	3.41	_	_	_	_	_	_	_
*QYr.uaf-3B.2*	IWB11270	3B	67.67	0.31	_	_	3.45	_	3.57	_	_	3.8	_	5.38
*QYr.uaf-3B.3*	IWB36652	3B	71.65	0.39	_	_	_	3.73	_	_	_	_	4.3	_
*QYr.uaf-3D.1*	IWA4725	3D	4.56	0.29	_	_	_	_	_	_	3.18	_	_	_
*QYr.uaf-4B.4*	IWB60835	4B	0	0.34	3.48	_	_	_	_	_	3.9	_	_	_
*QYr.uaf-4B.2*	IWA2031	4B	98.65	0.16	_	_	_	4.71	4.57	_	_	_	_	5.63
*QYr.uaf-5A.1*	IWB38719	5A	88.7	0.39	_	_	3.13	_	4.24	_	_	_	4.13	_
*QYr.uaf-5A.2*	IWA589	5A	123.21	0.25	_	_	_	3.12	4.87	_	_	_	8.34	7.07
*QYr.uaf-5B.2*	IWB9459	5B	110.56	0.25	_	_	4.2	_	_	_	_	_	_	_
*QYr.uaf-6A.4*	IWB29623	6A	40.6	0.32	_	_	3.23	_	_	_	_	4.25	_	_
*QYr.uaf-6B.2*	IWB26626	6B	113.67	0.39	_	_	_	_	_	_	_	_	_	5.32
*QYr.uaf-6D.2*	IWA6673	6D	9.47	0.38	3.05	_	_	_	_	_	_	_	_	_
*QYr.uaf-7A.1*	IWA4173	7A	218.7	0.48	_	_	_	_	3.56	3.15	_	_	_	_
*QYr.uaf-7B.3*	IWB22838	7B	73.79	0.22	_	_	_	3.58	_	_	_	_	6.3	_
*QYr.uaf-7B.4*	IWA6401	7B	77.73	0.34	_	_	_	_	_	_	_	_	_	5.12
*QYr.uaf-7D.1*	IWB26628	7D	144.96	0.44	_	_	_	_	_	_	_	_	_	3.33

^a^ Putative name of identified quantitative trait loci (QTL) from the marker–trait association, ^b^ SNP marker associated with all-stage resistance to stripe rust against different races, ^cd^ Chromosome number and the position of the associated SNP marker according to [25], ^e^ MAF: Minor allelic frequency, ^f^ *p*-value of marker–trait association with the IT scores of all locations, ^g^ *p*-value of the marker–trait association with SEV scores of all locations. PAK-UAF: University of Agriculture, Faisalabad, Pakistan; MTV: Mount Vernon, USA; PCFS: Palouse conservation field station, USA; BLUE: Best linear unbiased estimator.

## Supplementary Materials

The following are available online at https://www.mdpi.com/2223-7747/9/9/1056/s1. Table S1: Stripe rust infection type (IT) recording scale. Table S2: Virulence and avirulence formula for the USA stripe rust races. Table S3: Virulence and avirulence formula for the Pakistan stripe rust. Table S4: ΔK value based on LnP(K) for 465 wheat genotypes. Figures S1 and S3: Manhattan representing the number of chromosome and their associated SNPs. Figures S2 and S4: Q-Q plot representing the number of chromosome and their associated SNPs.

## Abbreviations

Pst	*Puccinia striiformis* f. sp. *tritici*
MH	Million hectare
Yr	Yellow rust
USA	United State of America
APR	Adult plant resistance
MAS	Marker assisted selection
LD	Linkage disequilibrium
AM	Association mapping
GWAS	Genome-wide association mapping
QTL	Quantitative trait loci
SNP	Single nucleotide polymorphism
SEV	Severity
DArT	Diversity Arrays Technology
SSR	Simple sequence repeats
PCFS	Palouse conservation field station
MTV	Mount Vernon
UAF	University of Agriculture, Faisalabad
AvS	Avocet susceptible
MAF	Minor allelic frequency
TAS	Targeted amplicon sequencing
MLM	Mixed linear model
MTA	Marker–trait association
TASSEL	Trait analysis by association, evolution and linkage
