# Supplementary material for: Genome-Wide Association Mapping for Stripe Rust Resistance in Pakistani Spring Wheat Genotypes
Source: Plants (Basel). 2020 Aug 19;9(9):1056. doi: 10.3390/plants9091056 (PMC7570266; doi:10.3390/plants9091056)
Supplement: Supplementary file 1 [file plants-09-01056-s001.zip › plants-818336-xml01/plants-818336-Supplementary Figures S1,S2, S3, S4.docx]

| **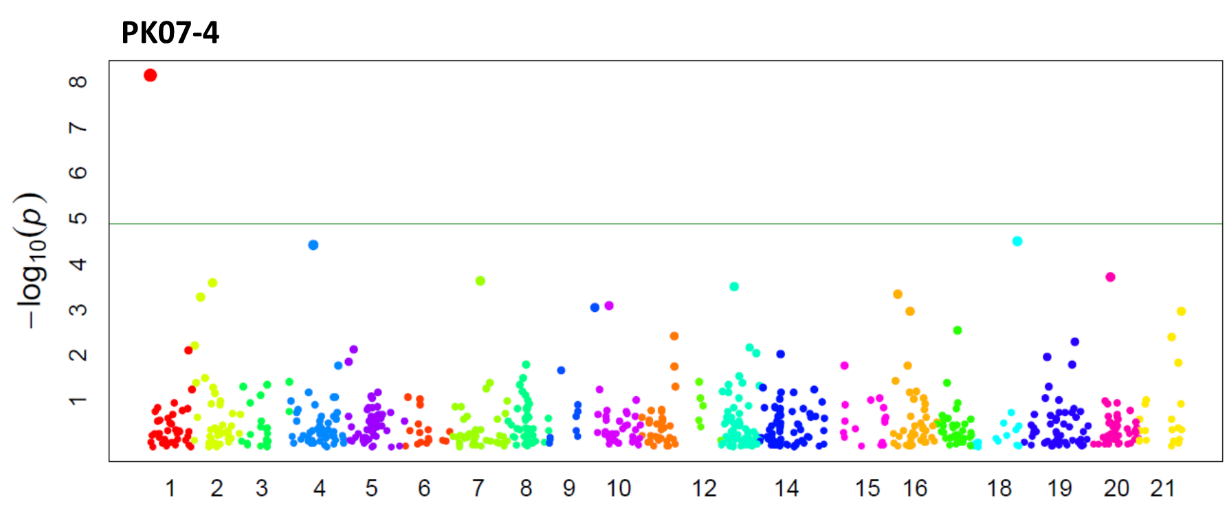** |
| --- |
| (**A**) Manhattan plot of race PK07-4 |
| **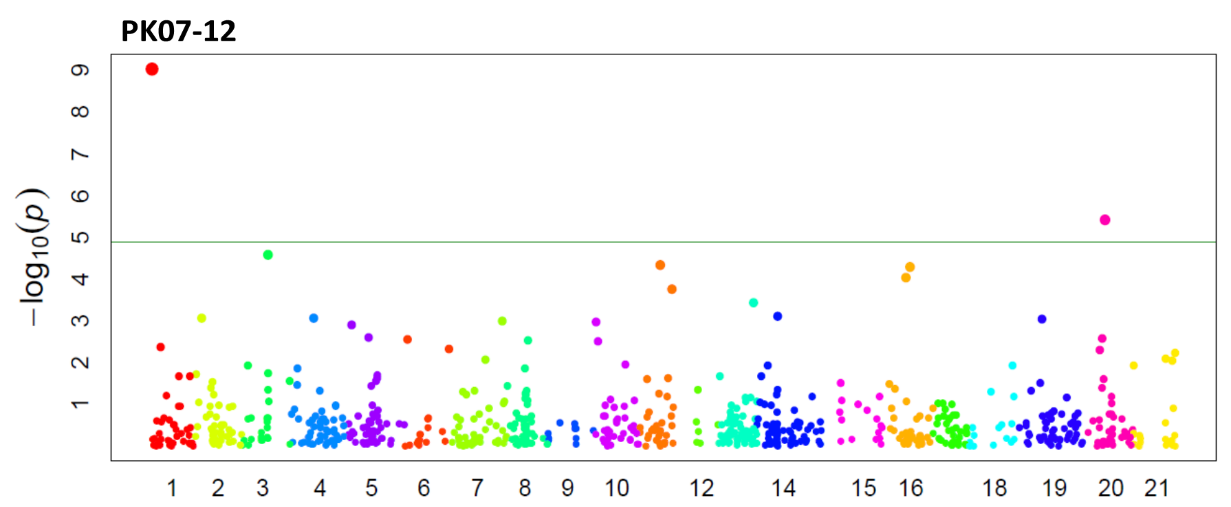** |
| (**B**) Manhattan plot of race PK07-12 |
| **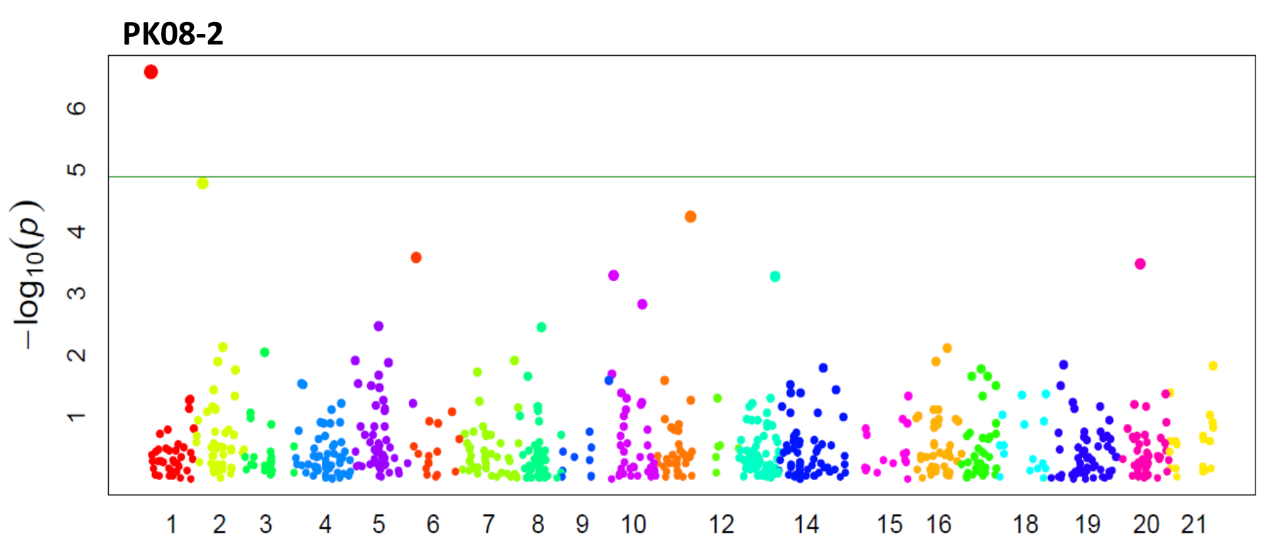** |
| (**C**) Manhattan plot of race PK08-2 |
| **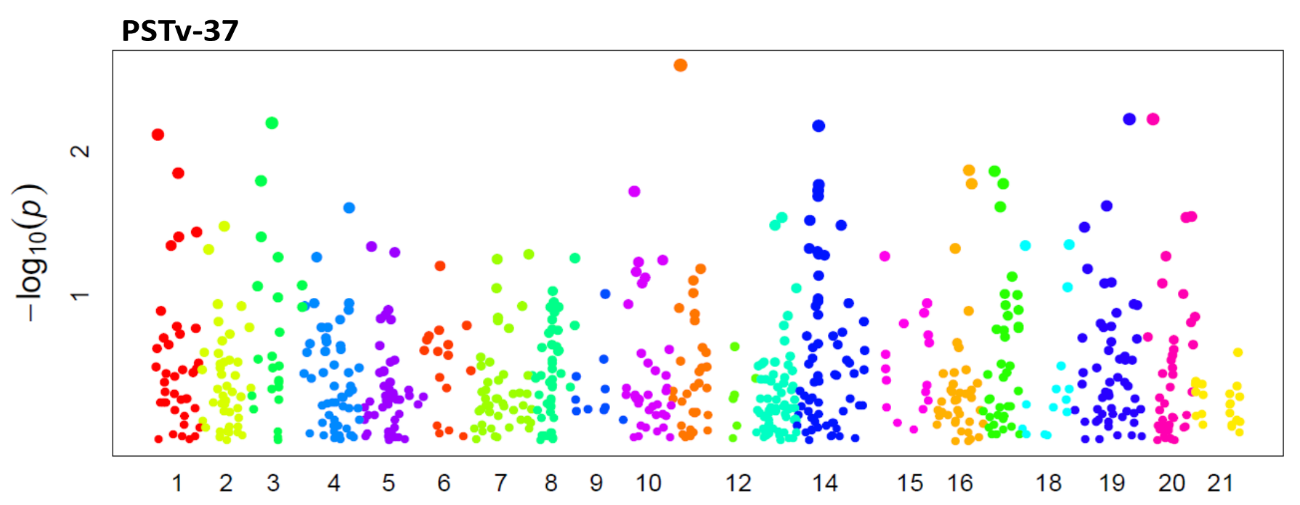** |
| (**D**) Manhattan plot of race PSTv-37 |
| 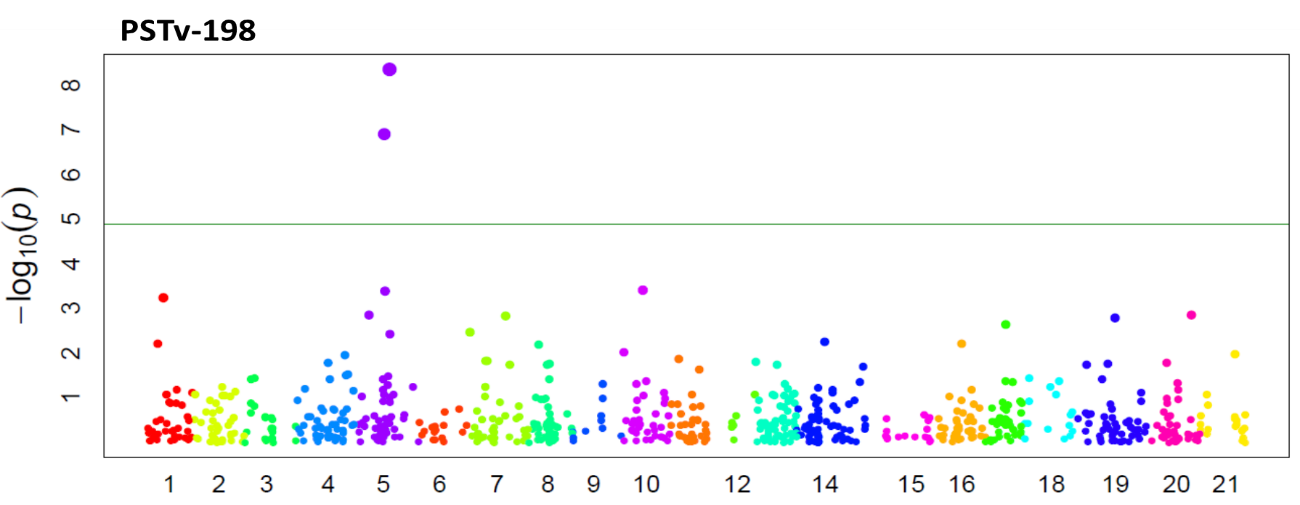 |
| (**E**) Manhattan plot of race PSTv-198 |
| 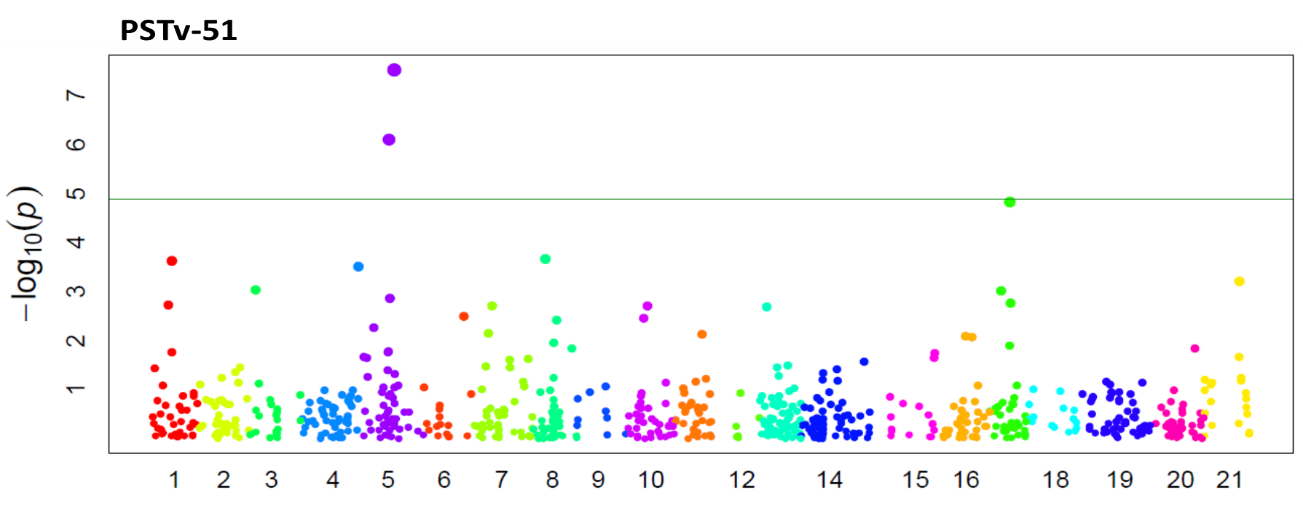 |
| (**F**) Manhattan plot of race PSTv-51 |
| 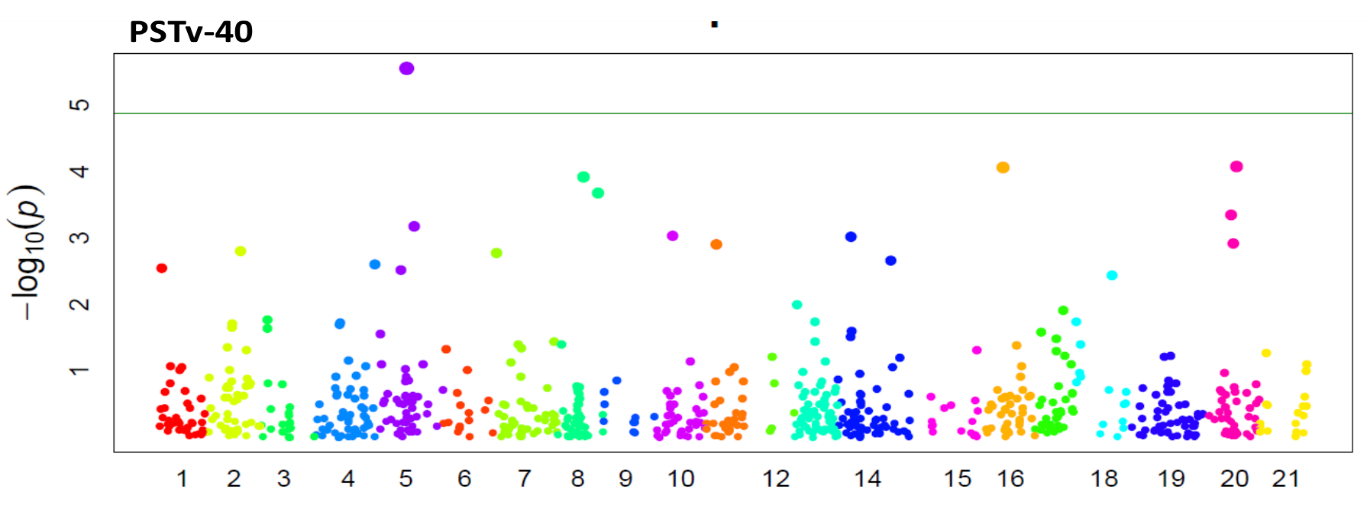 |
| (**G**) Manhattan plot of race PSTv-40 |
| 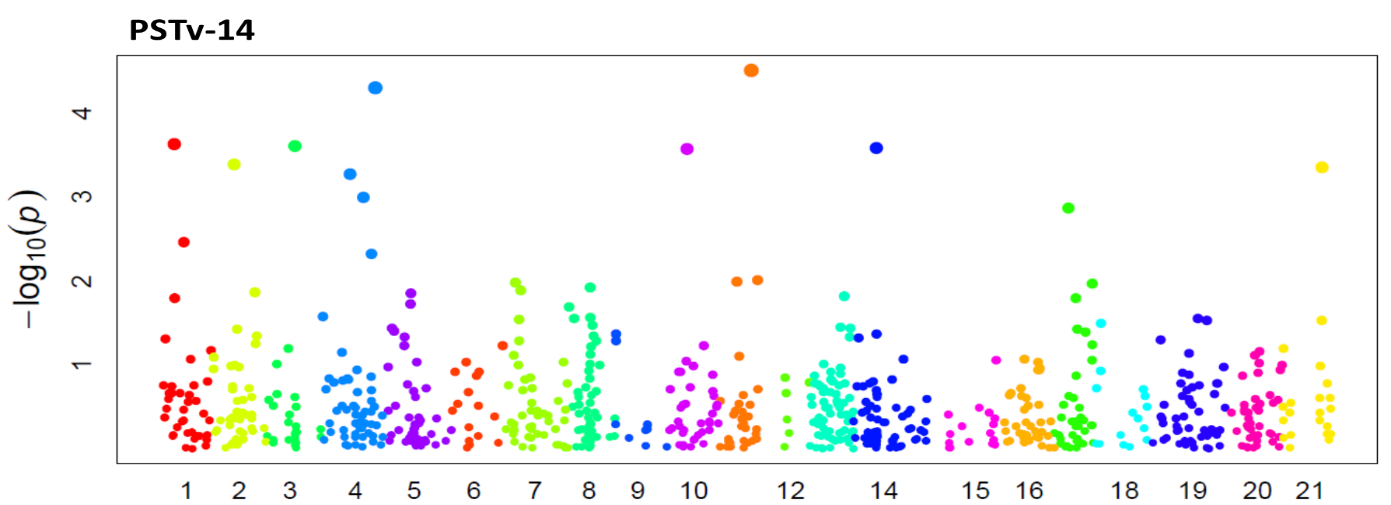 |
| (**H**) Manhattan plot of race PSTv-14 |
| **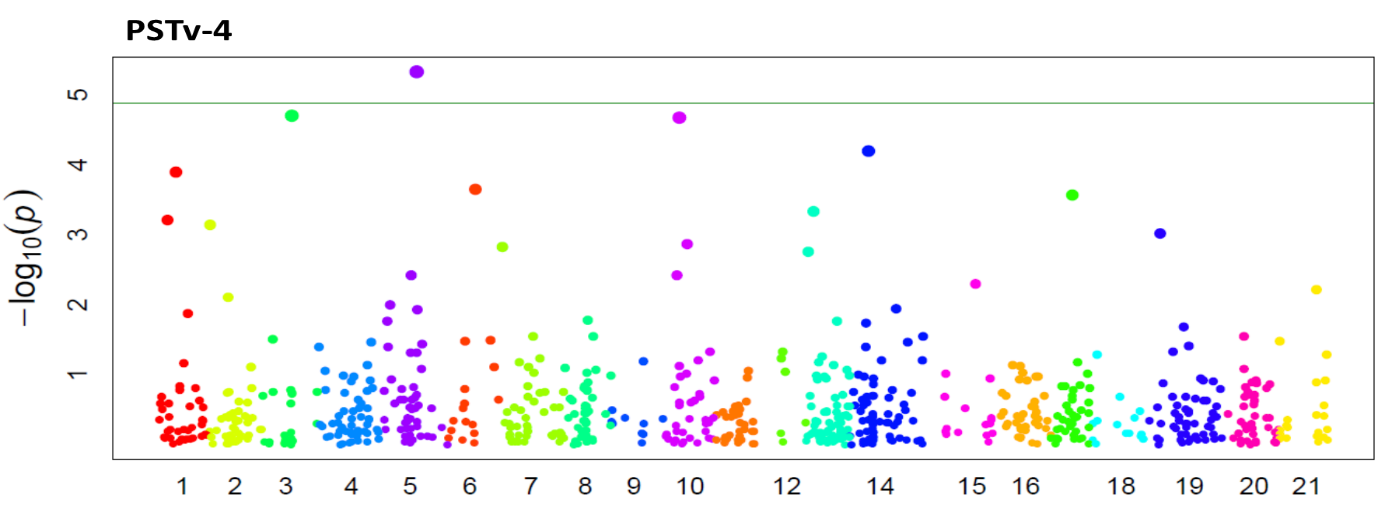** |
| (**I**) Manhattan plot of race PSTv-4 |

**Figure S1.** Manhattan representing the number of chromosome and their associated SNPs. **A:** PK07-4 **B:** PK07-12 **C:** PK08-2 **D:** PSTv-37 **E:** PSTv-198 **F:** PSTv-51 **G:** PSTv-40 **H:** PSTv-14 **I:** PSTv-4.

| 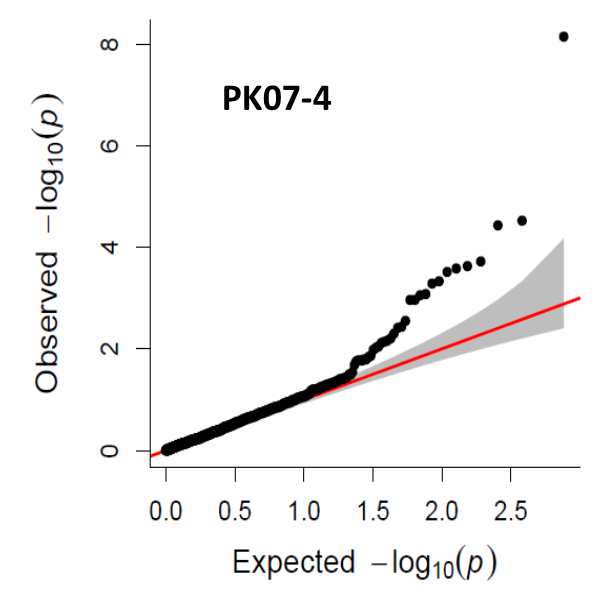 | 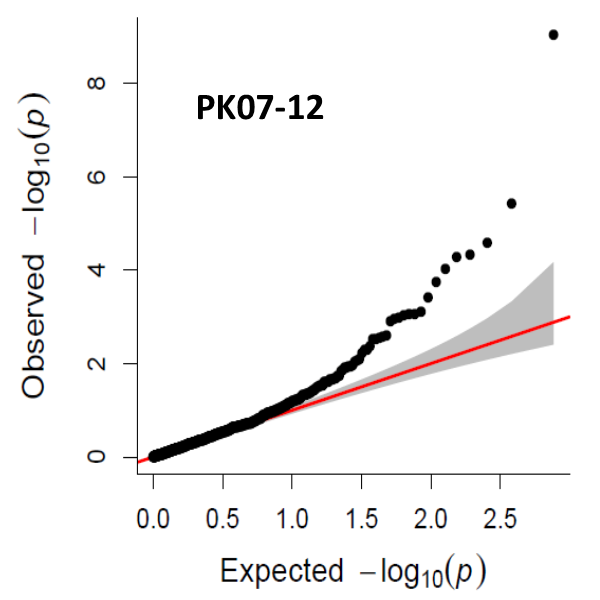 |
| --- | --- |
| A | B |
| 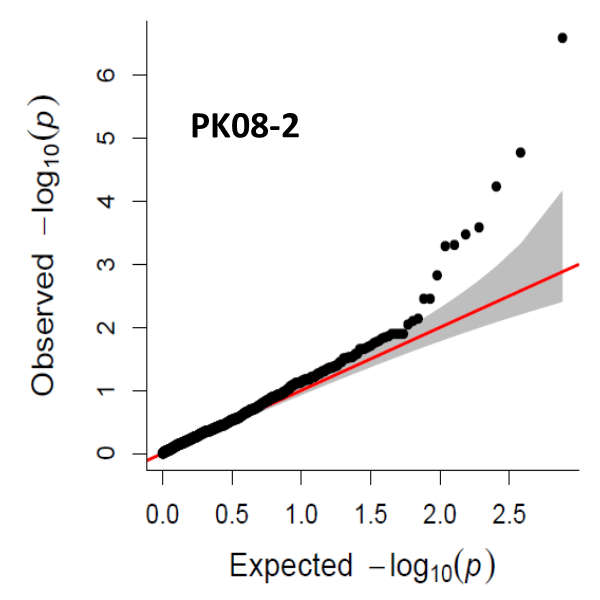 | 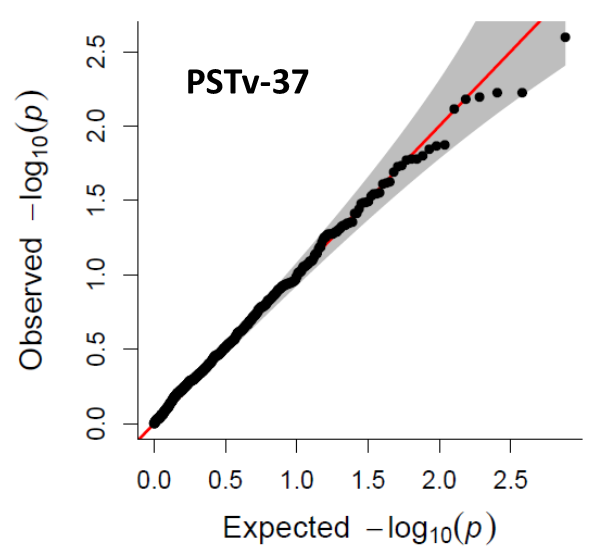 |
| C | D |
| 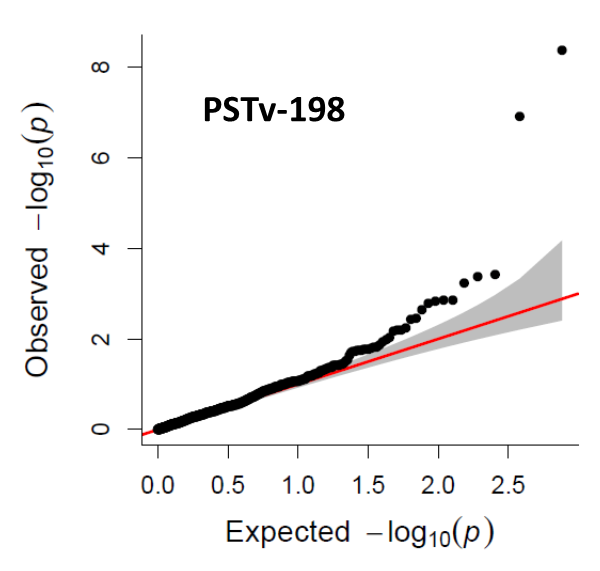 | 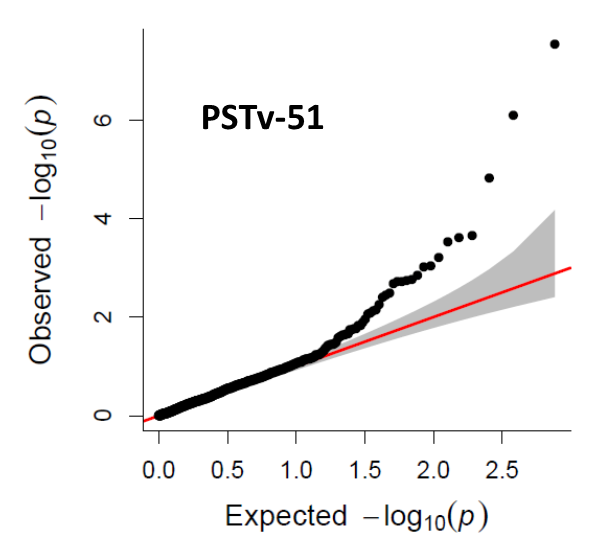 |
| E | F |
| 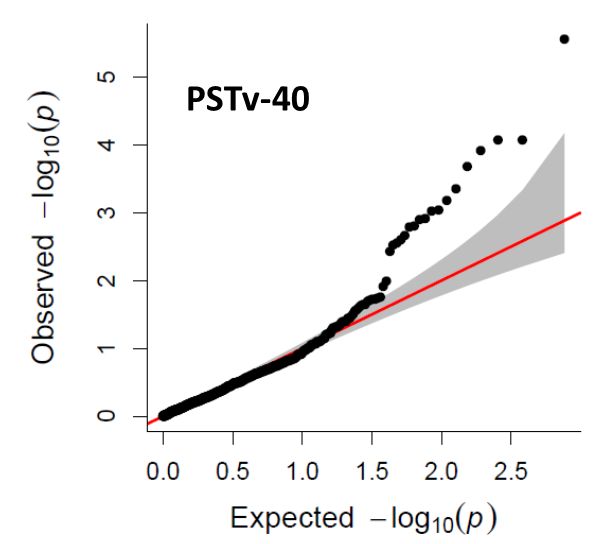 | 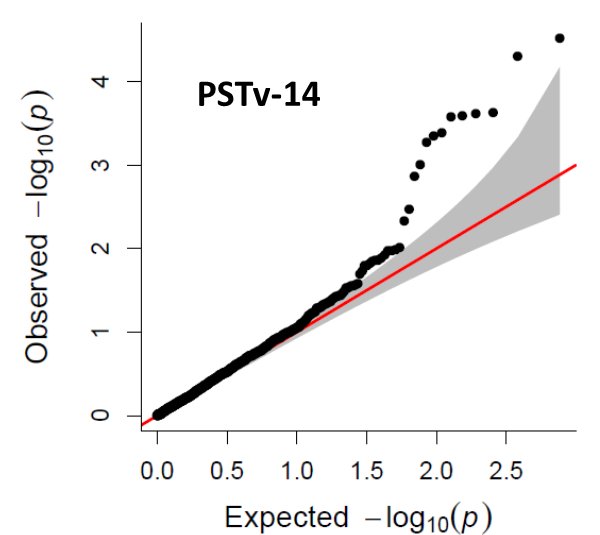 |
| G | H |
| 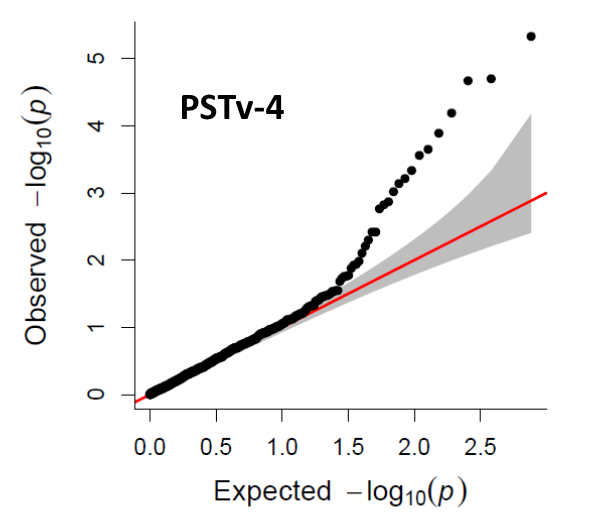 |  |
| I |  |

**Figure S2.** QQ plot representing the number of chromosome and their associated SNPs. **A:** PK07-4 **B:** PK07-12 **C:** PK08-2 **D:** PSTv-37 **E:** PSTv-198 **F:** PSTv-51 **G:** PSTv-40 **H:** PSTv-14 **I:** PSTv-4.

| **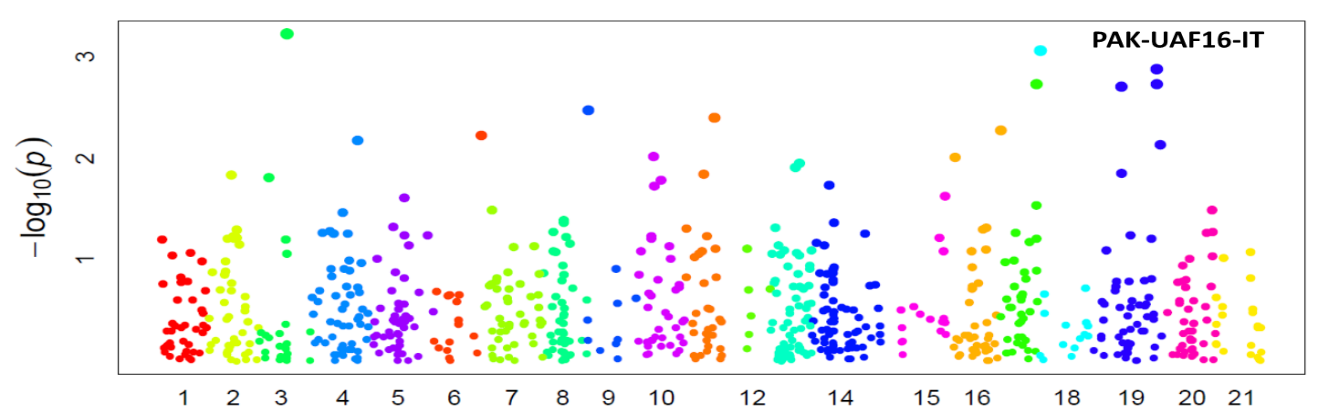** | (A) |
| --- | --- |
| 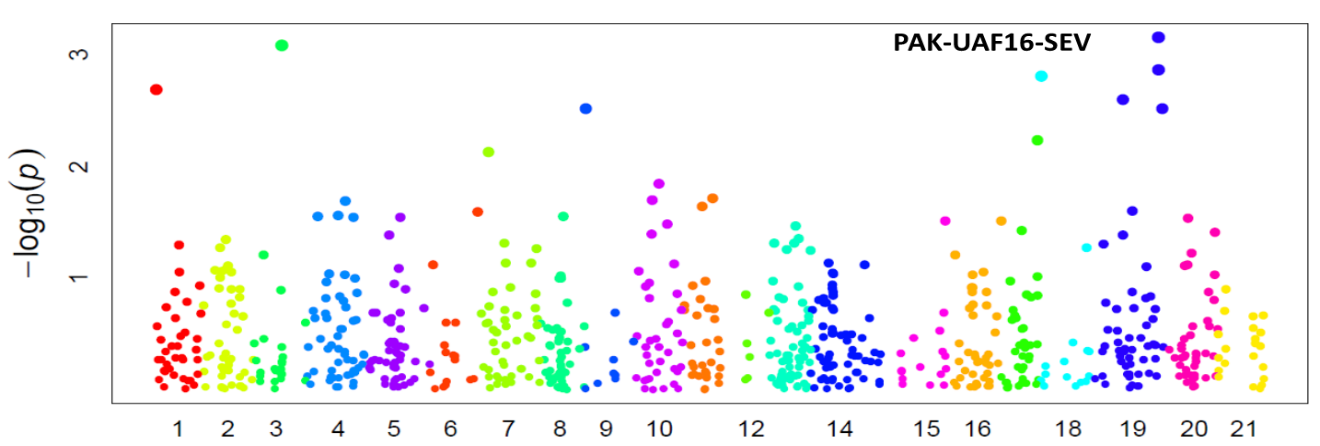 | (B) |
| 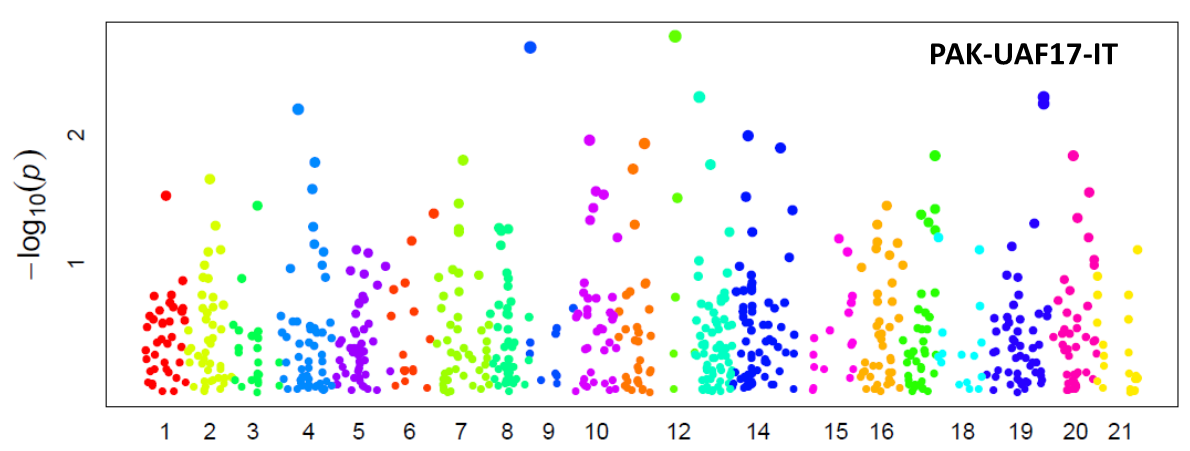 | (C) |
| 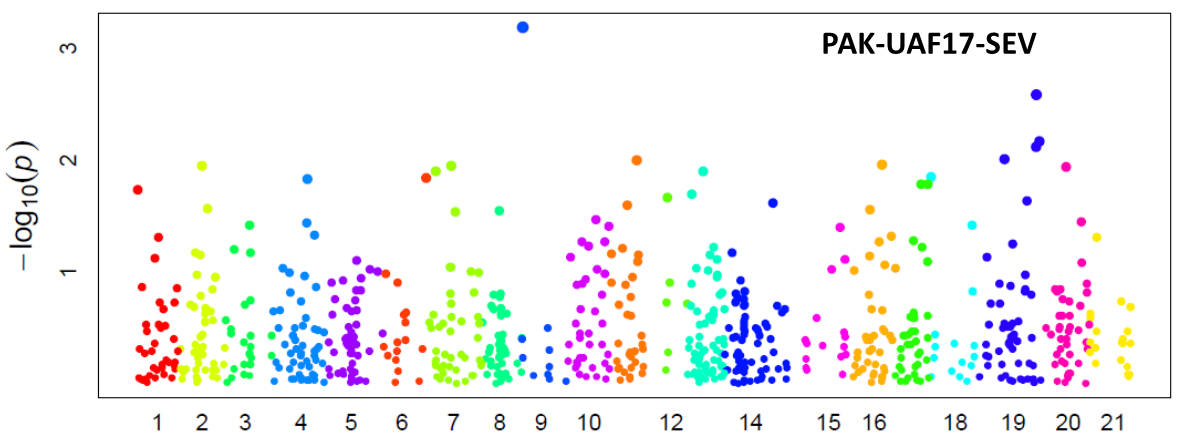 | (D) |
| 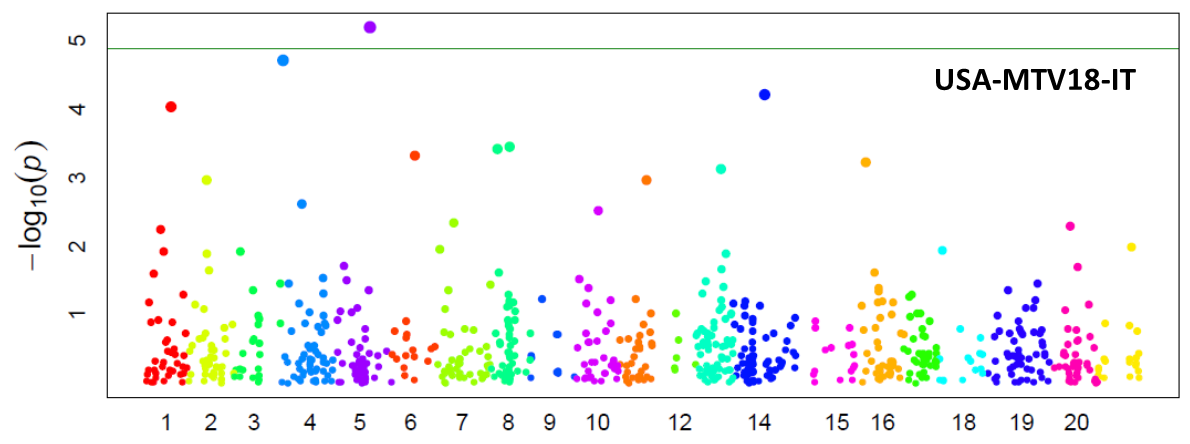 | (E) |
| 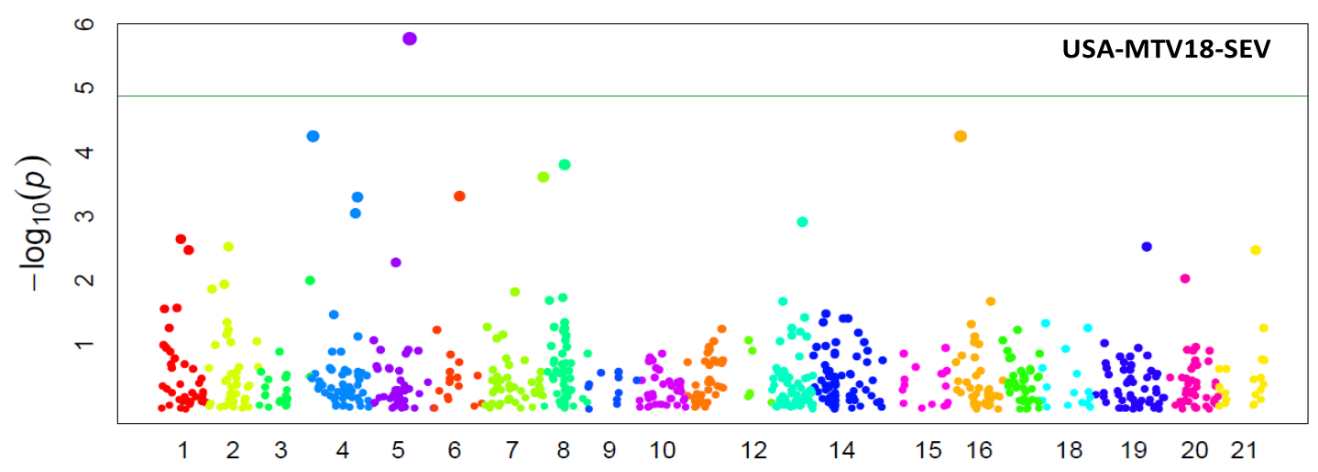 | (F) |
| 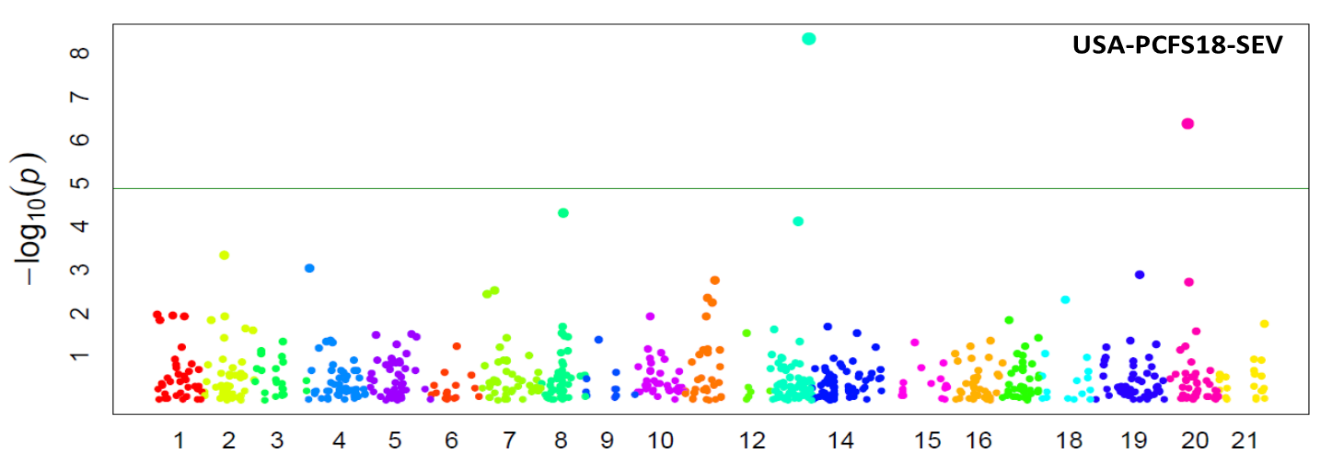 | （G) |
| 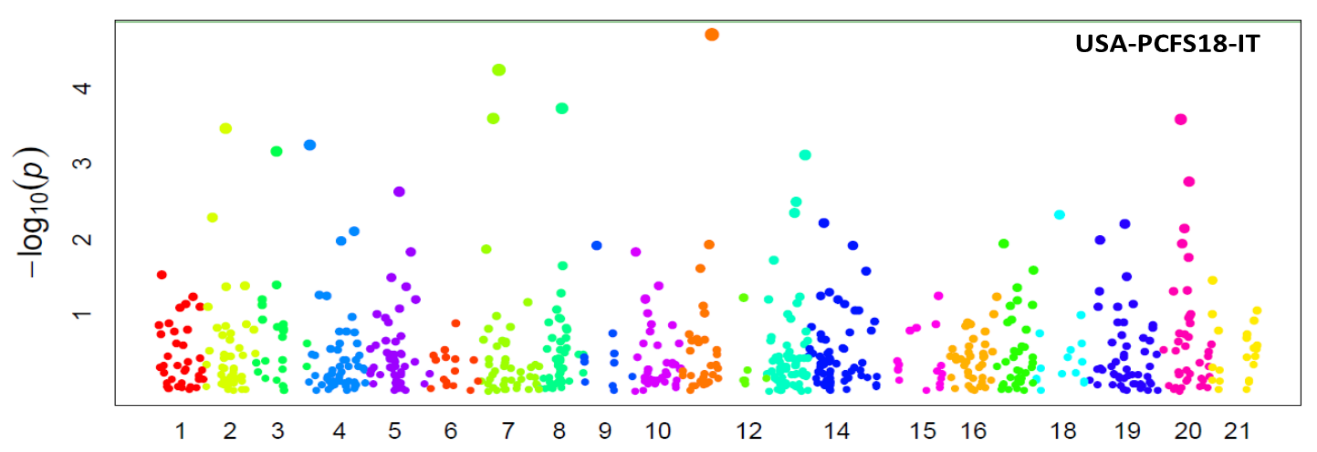 | （H） |
| **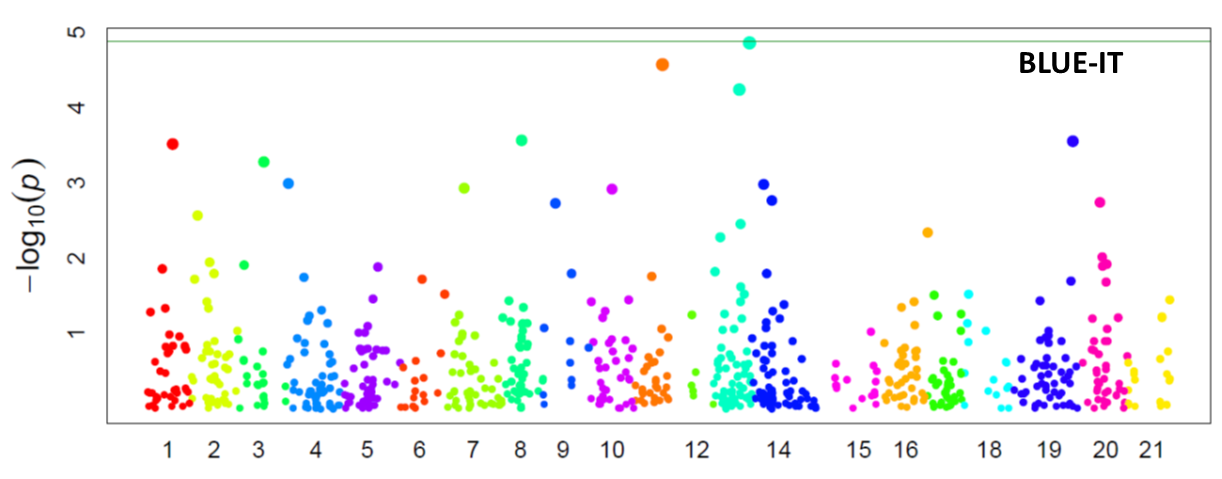** | （I） |
| 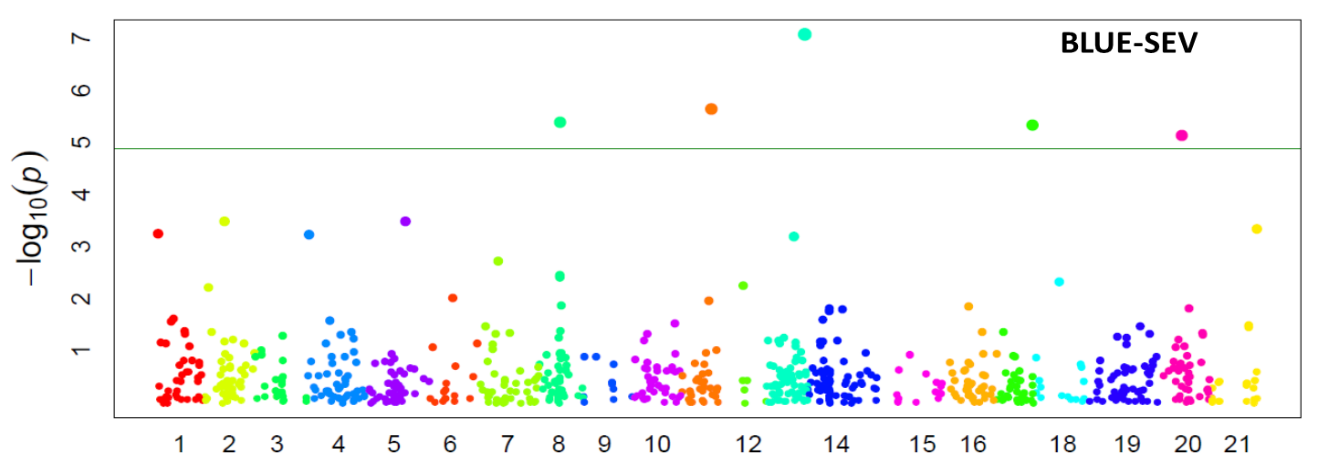 | （J） |

**Figure S3.** Manhattan representing the number of chromosome and their associated SNPs. **(A):** PAK-UAF16 (IT) **(B):** PAK-UAF16 (SEV) **(C):** PAK-UAF17 (IT) **(D):** PAK-UAF17 (SEV) **(E):** USA-MTV18 (IT) **(F):** USA-MTV18 (SEV) (**G):** USA-PCFS18 (IT) **(H):** USA-PCFS18 (SEV) **(I):** BLUE-IT **(J):** BLUE-SEV.

| 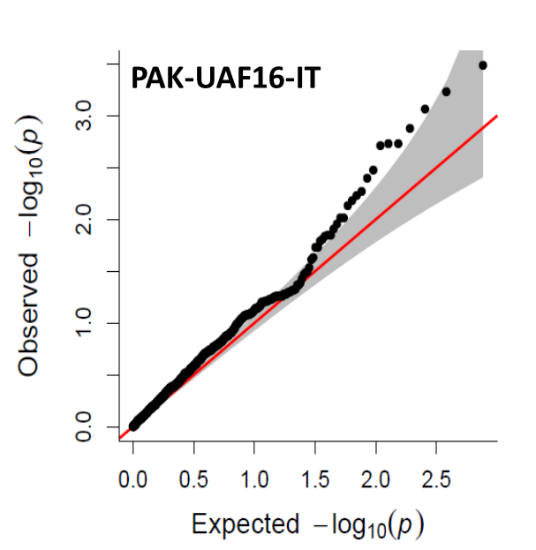 | 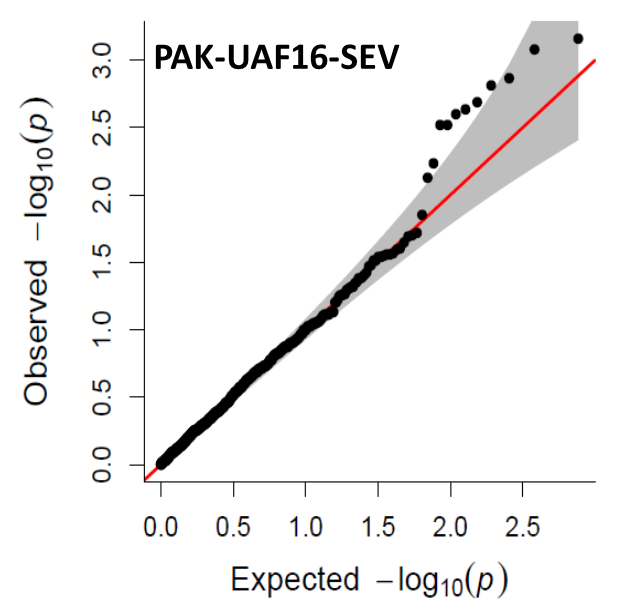 |
| --- | --- |
| (**A**) | (**B**) |
| 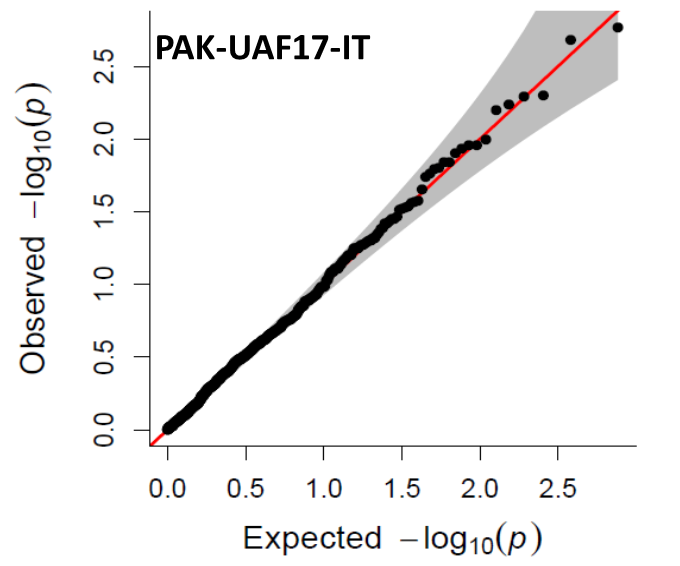 | 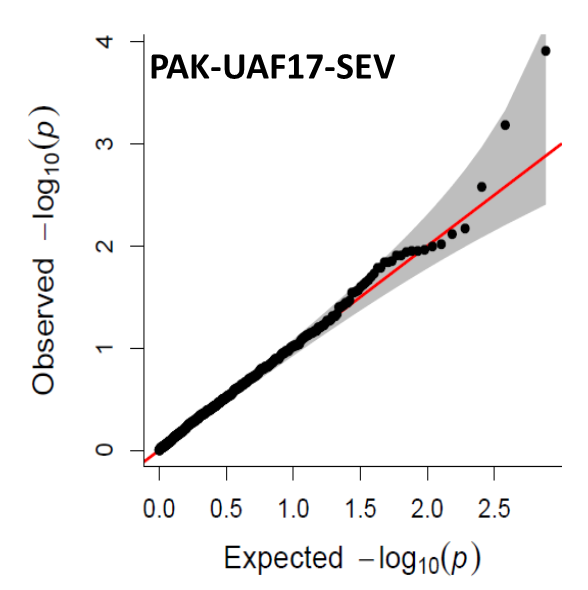 |
| (**C**) | (**D**) |
| 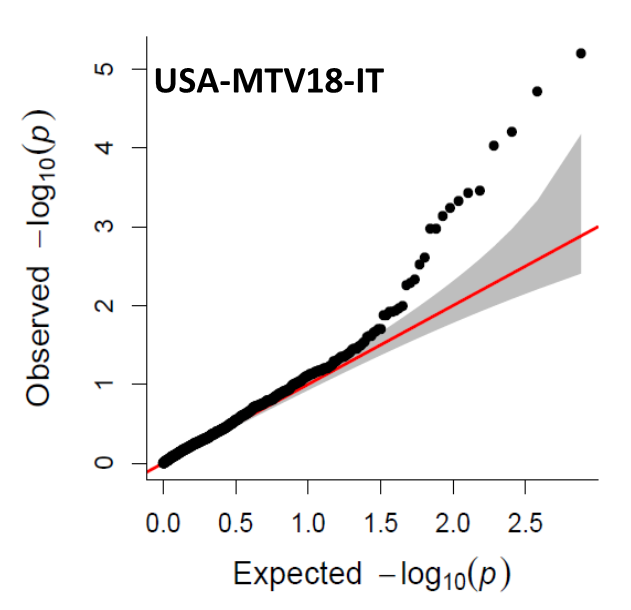 | 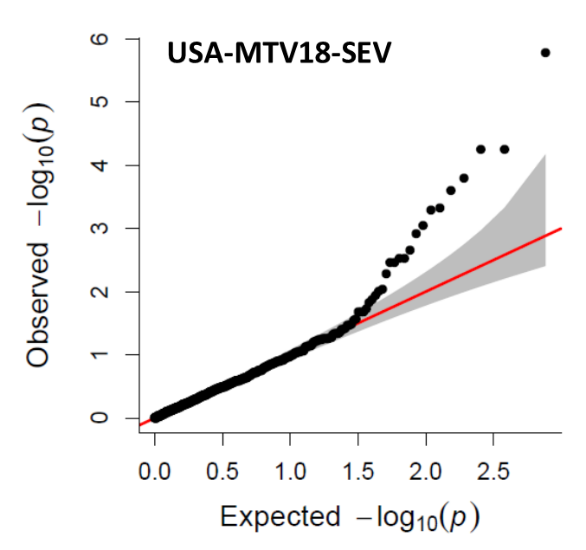 |
| (**E**) | (**F**) |
| 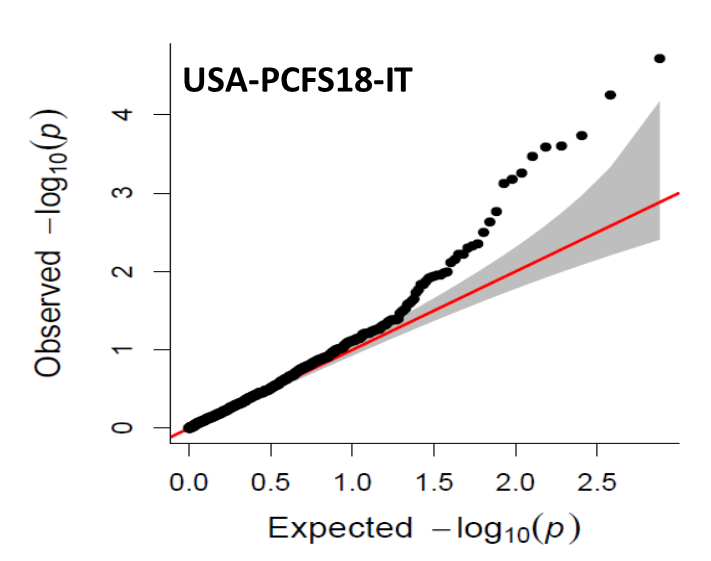 | 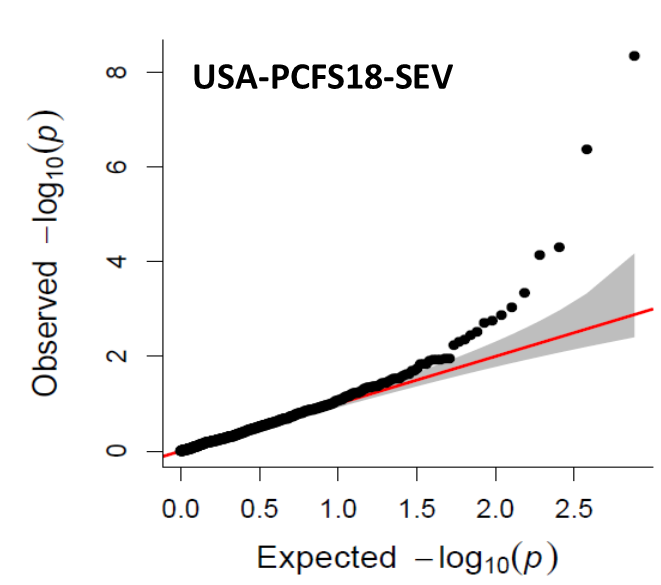 |
| (**G**) | (**H**) |
| 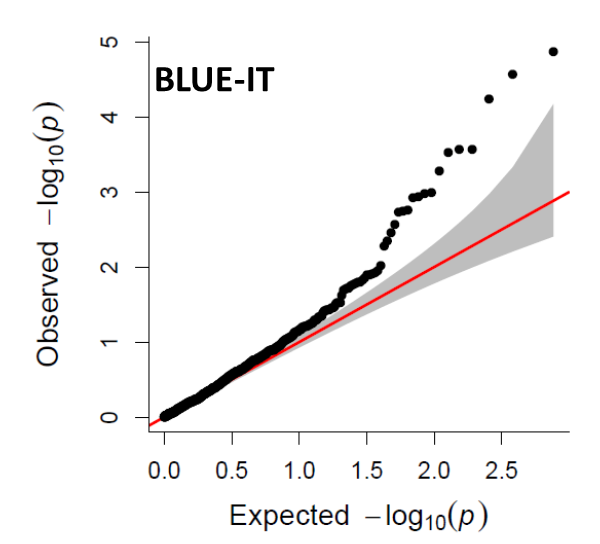 | 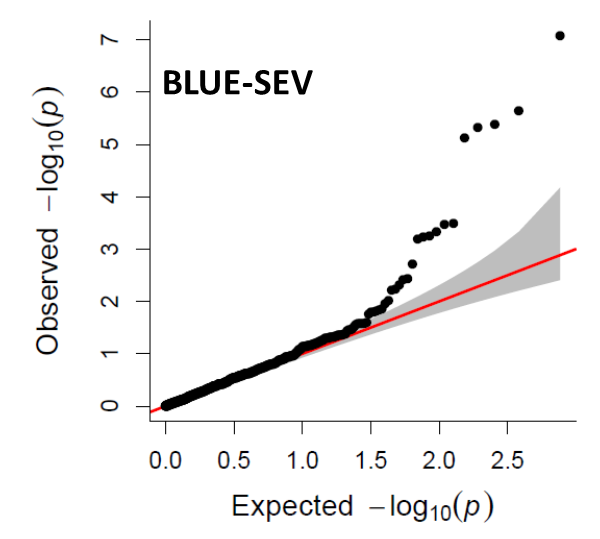 |
| (**I**) | (**J**) |

**Figure S4.** QQ plot representing the number of chromosome and their associated SNPs. **(A):** PAK-UAF16 (IT) **(B):** PAK-UAF16 (SEV) **(C):** PAK-UAF17 (IT) **(D):** PAK-UAF17 (SEV) **(E):** USA-MTV18 (IT) **(F):** USA-MTV18 (SEV) (**G):** USA-PCFS18 (IT) **(H):** USA-PCFS18 (SEV) **(I):** BLUE-IT **(J):** BLUE-SEV.
